# Supplementary material for: Dietary-Derived Essential Nutrients and Amyotrophic Lateral Sclerosis: A Two-Sample Mendelian Randomization Study
Source: Nutrients. 2022 Feb 22;14(5):920. doi: 10.3390/nu14050920 (PMC8912818; doi:10.3390/nu14050920)
Supplement: Supplementary file 1 [file nutrients-14-00920-s001.zip › supplementary materials/additional file 2 Supplementary Figure 1-2.pdf]

# Dietary derived essential nutrients and amyotrophic lateral sclerosis: a two-sample mendelian randomization study

Kailin Xia[1,2,3#], Yajun Wang[1,2,3#], Linjing Zhang[1,2,3], Lu Tang[1,2,3], Gan Zhang[1,2,3], Tao Huang[4], Ninghao Huang[4], Dongsheng Fan[1,2,3*]

[1]Department of Neurology, Peking University Third Hospital, Beijing, China.

[2]Beijing Key Laboratory of Biomarker and Translational Research in Neurodegenerative Diseases, Beijing, China

[3]Key Laboratory for Neuroscience, National Health Commission/Ministry of Education, Peking University, Beijing, China

[4]Department of Epidemiology and Biostatistics, School of Public Health, Peking University, Beijing, China

#KX and YW contributed equally to this article.

*Corresponding author: Dr. Dongsheng Fan**,**

Department of Neurology, Peking University Third Hospital,

49 North Garden Road, Haidian District, Beijing 100191, People's Republic of China.

E-mail:dsfan2010@aliyun.com

# Supplemental Figures

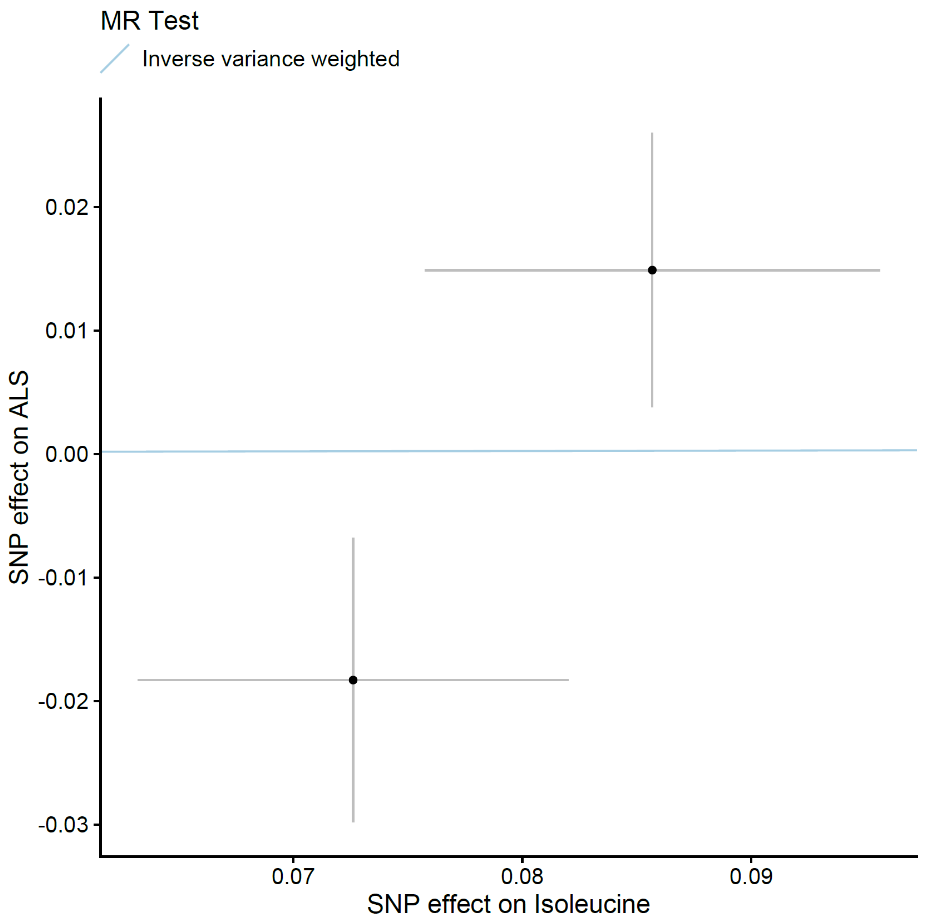


Supplemental Figure S1A. Scatterplot of isoleucine and amyotrophic lateral sclerosis

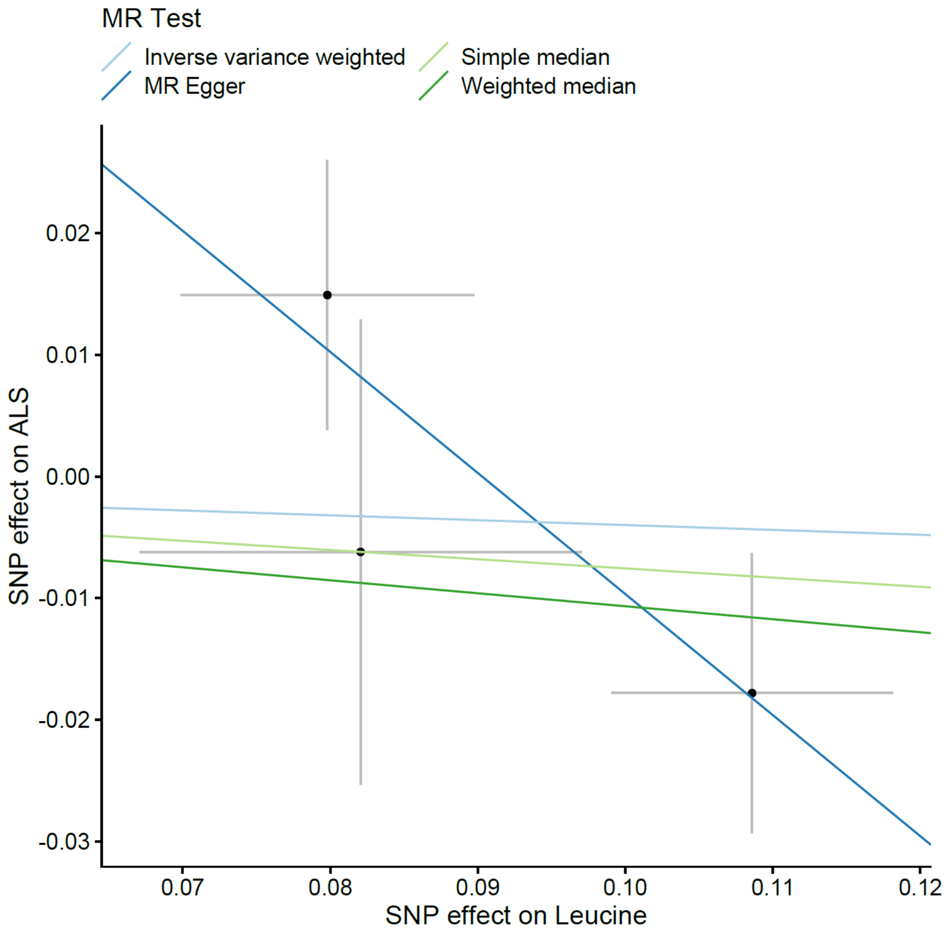


Supplemental Figure S1B. Scatterplot of leucine and amyotrophic lateral sclerosis

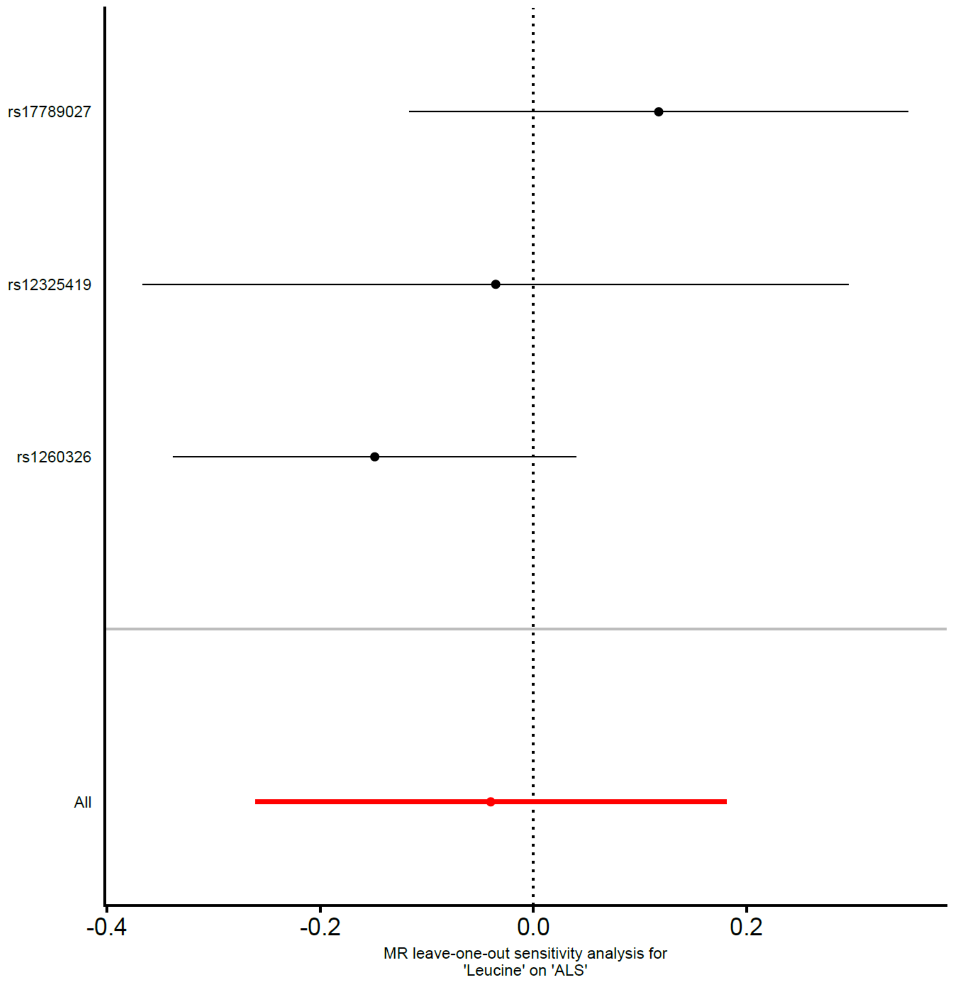


Supplemental Figure S1C Forrest plot of leucine and amyotrophic lateral sclerosis

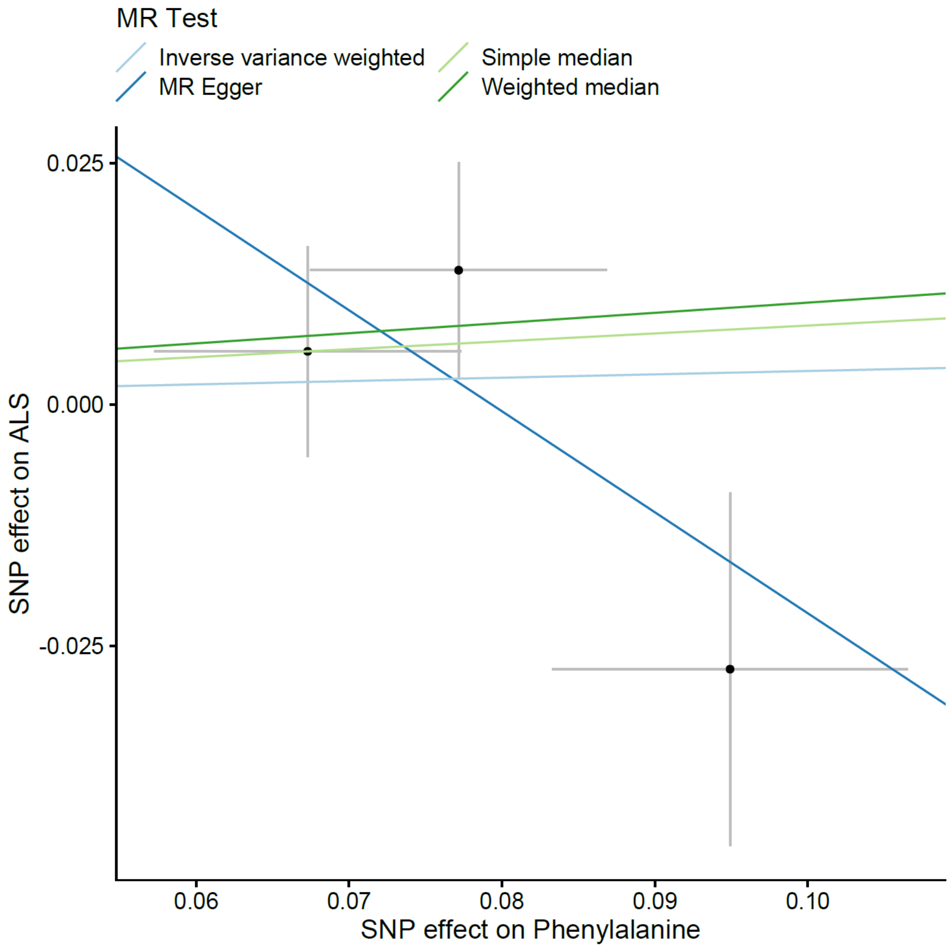


Supplemental Figure S1D. Scatterplot of phenylalanine and amyotrophic lateral sclerosis

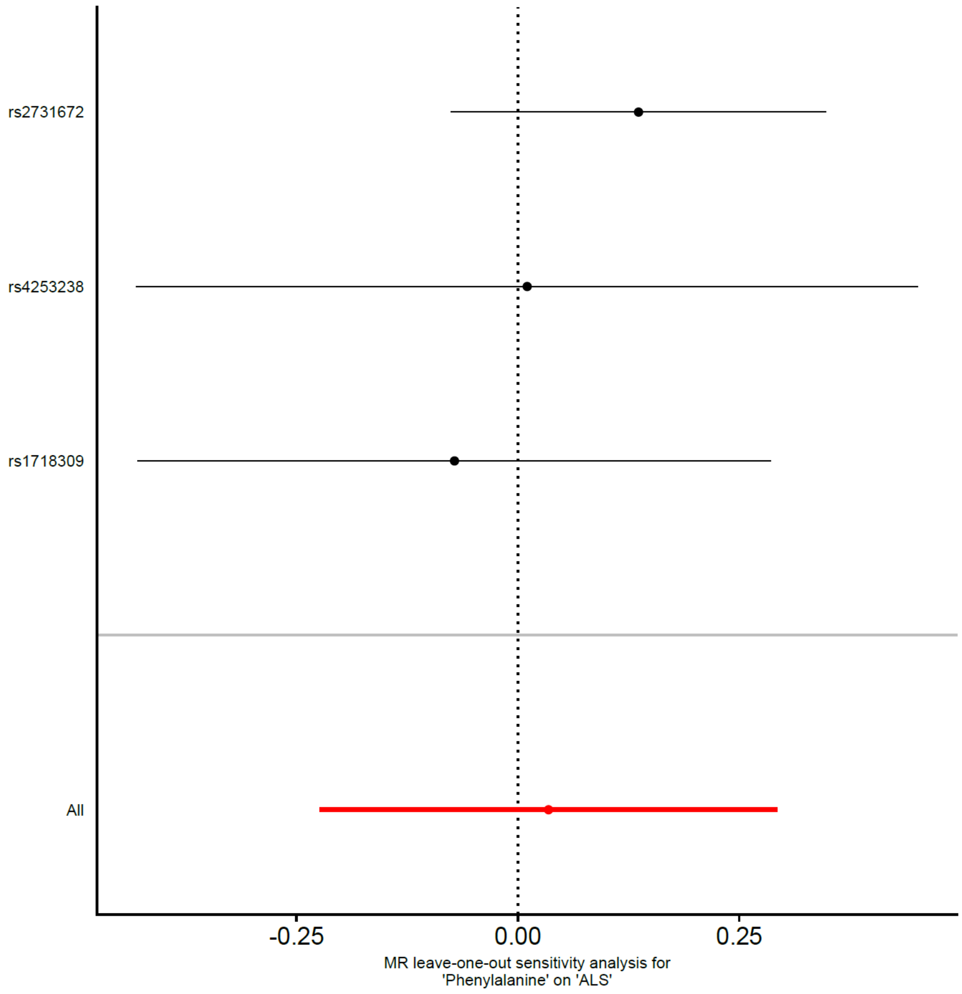


Supplemental Figure S1E. Forrest plot of phenylalanine and amyotrophic lateral sclerosis

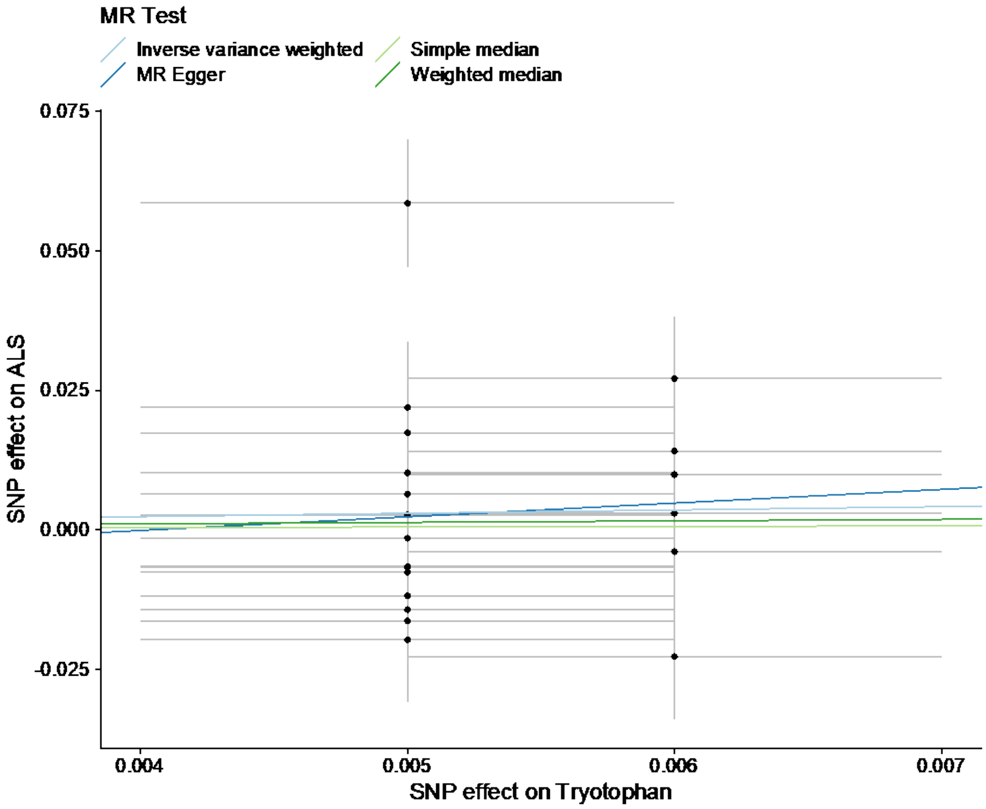


Supplemental Figure S1F. Scatterplot of tryotophan and amyotrophic lateral sclerosis

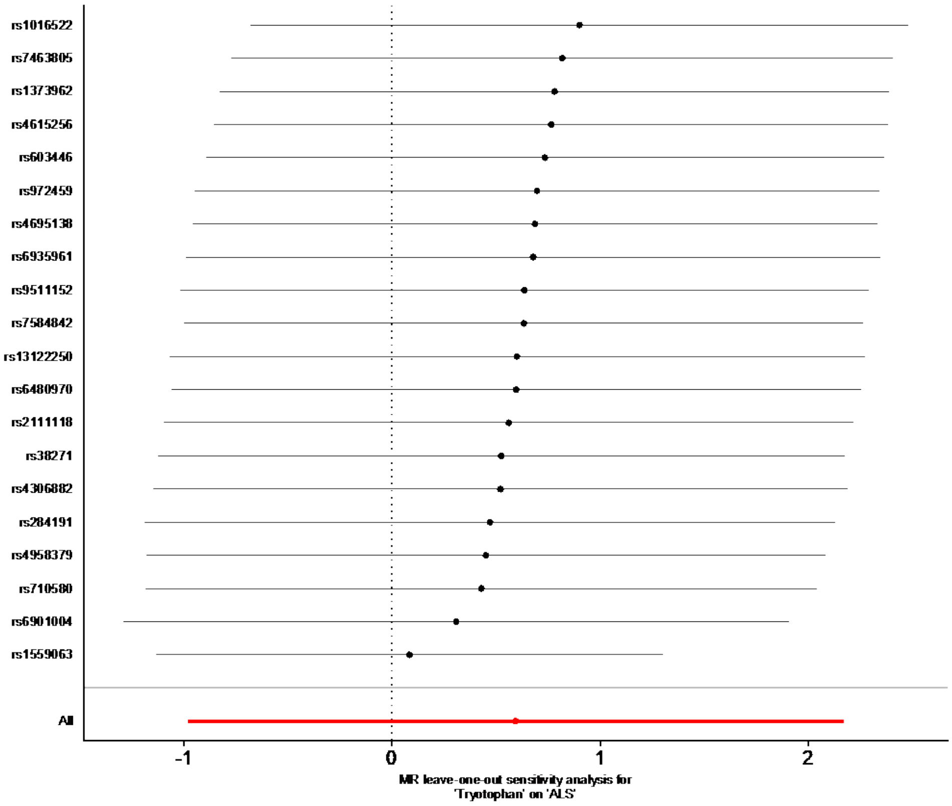


Supplemental Figure S1G. Forrest plot of tryotophan and amyotrophic lateral sclerosis

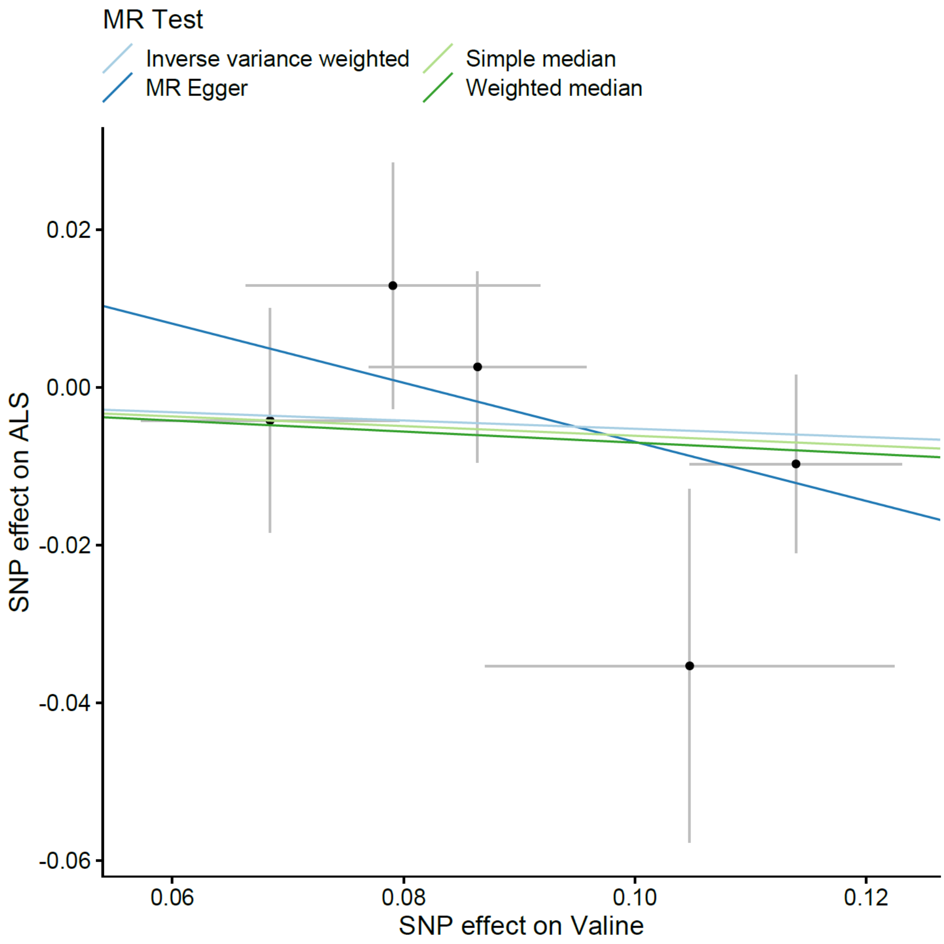


Supplemental Figure S1H. Scatterplot of valine and amyotrophic lateral sclerosis

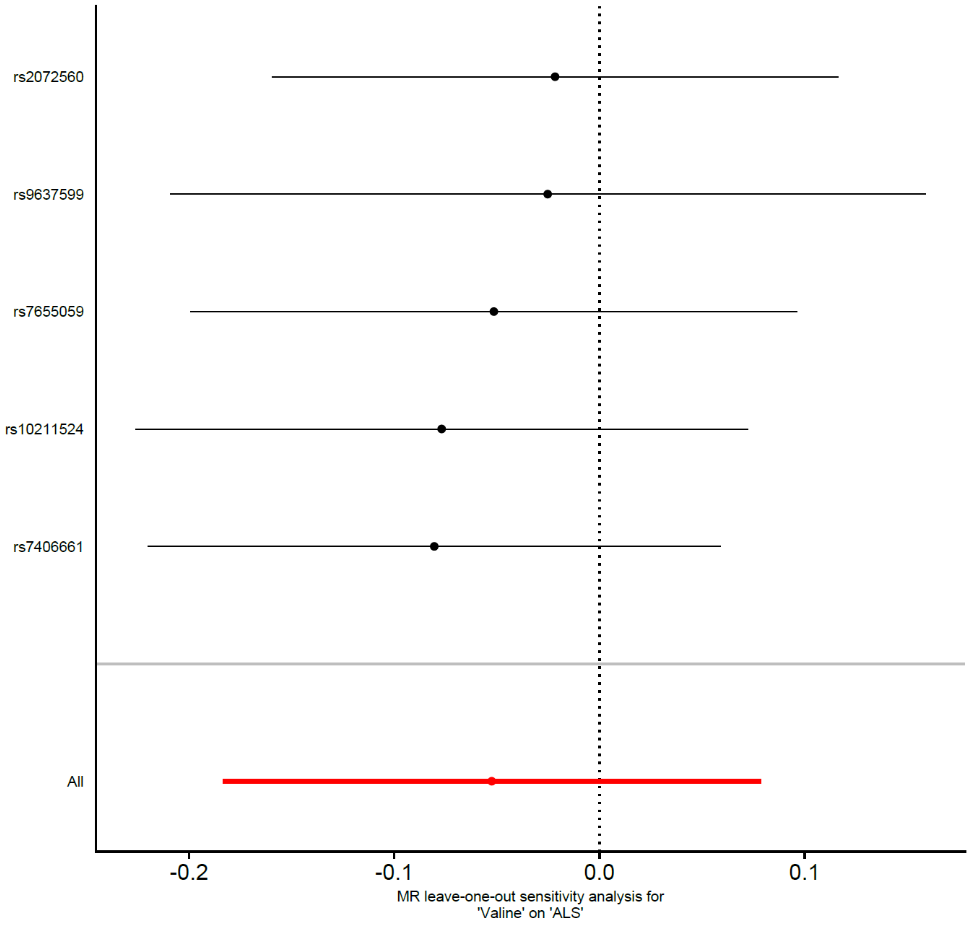


Supplemental Figure S1I. Forrest plot of valine and amyotrophic lateral sclerosis

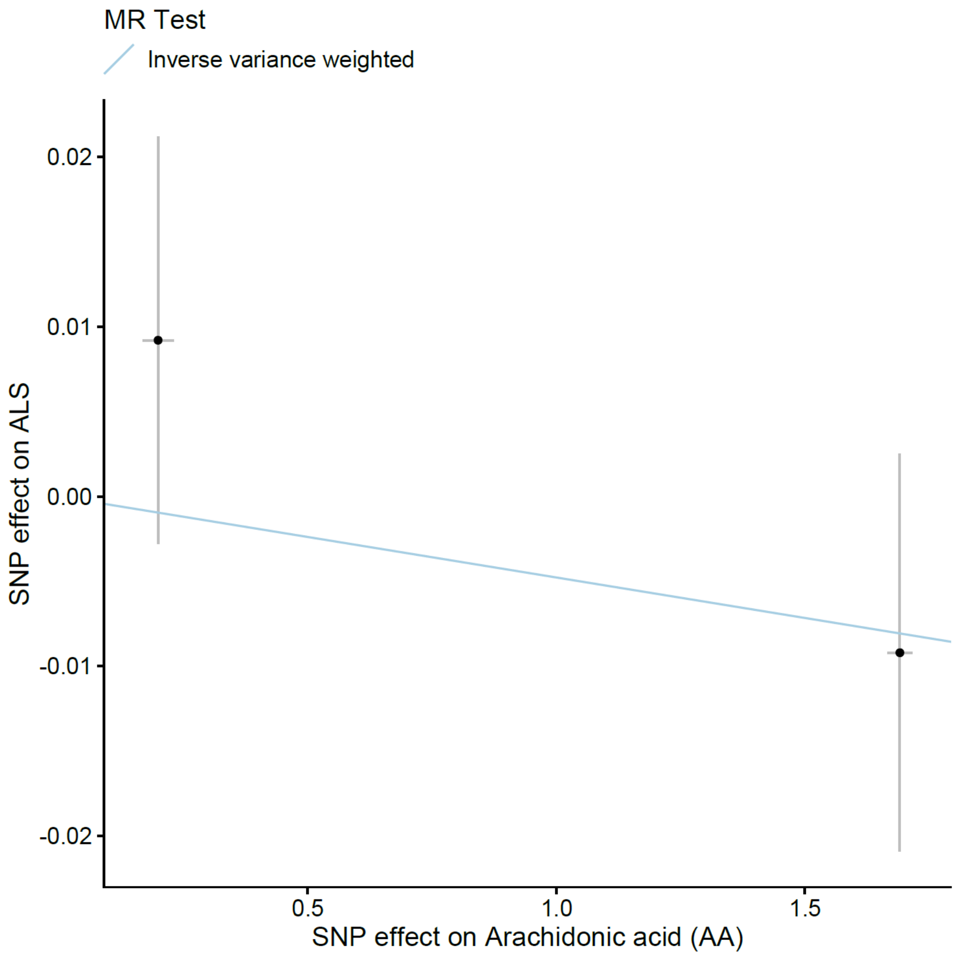


Supplemental Figure S2A. Scatterplot of arachidonic acid (AA) and amyotrophic lateral sclerosis

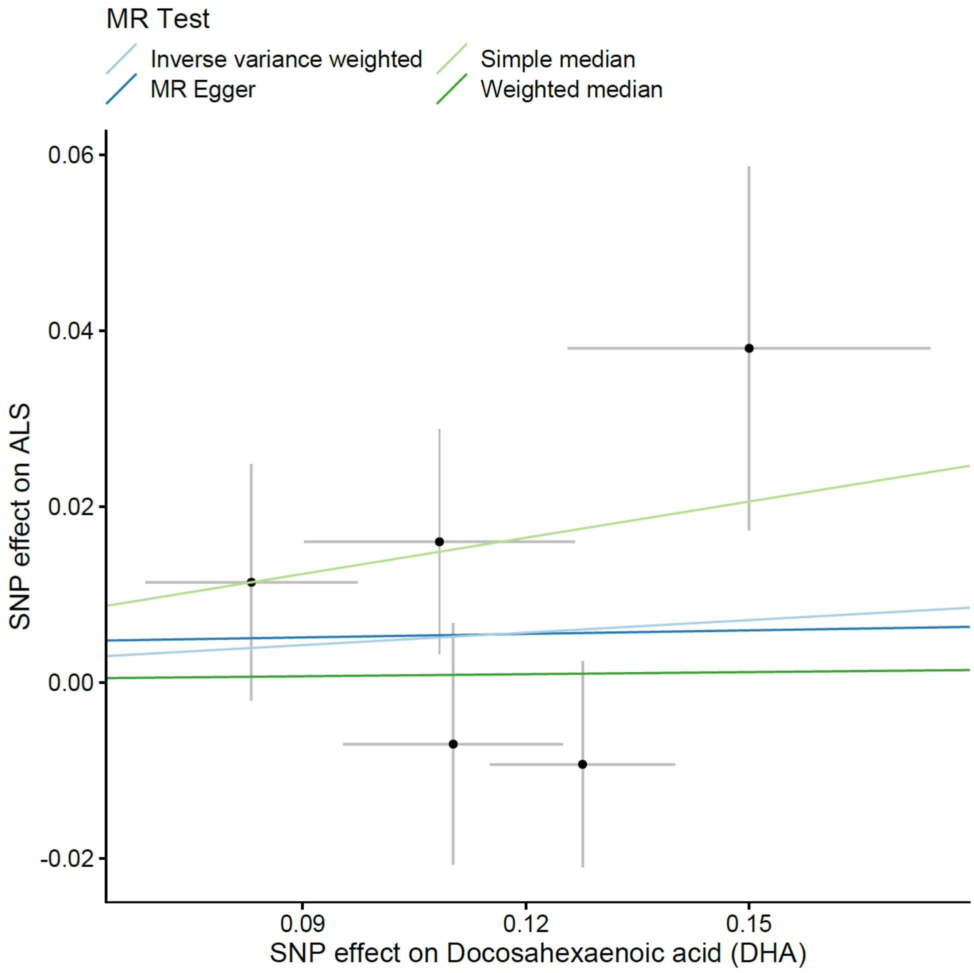


Supplemental Figure S2B. Scatterplot of docosahexaenoic (DHA) and amyotrophic lateral sclerosis

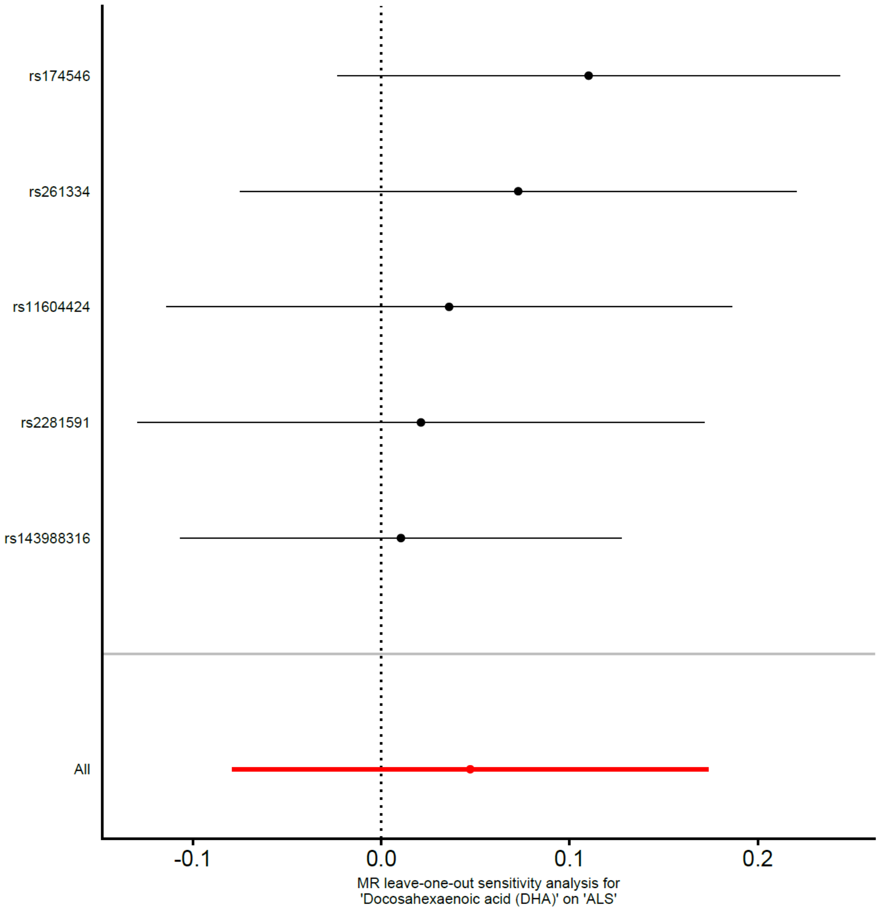


Supplemental Figure S2C. Forrest plot of docosahexaenoic (DHA) and amyotrophic lateral sclerosis

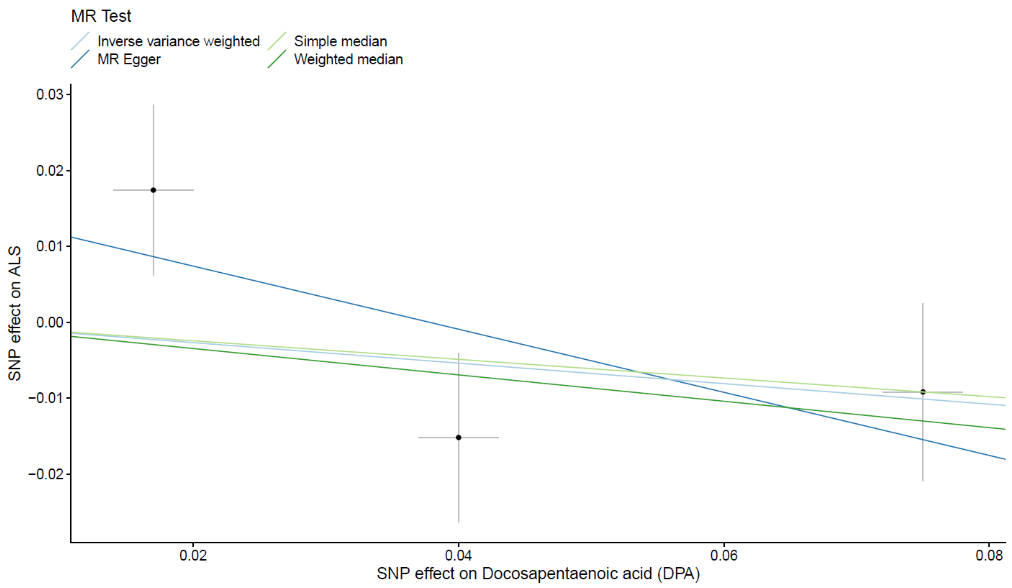


Supplemental Figure S2D. Scatterplot of docosapentaenoic (DPA) and amyotrophic lateral sclerosis

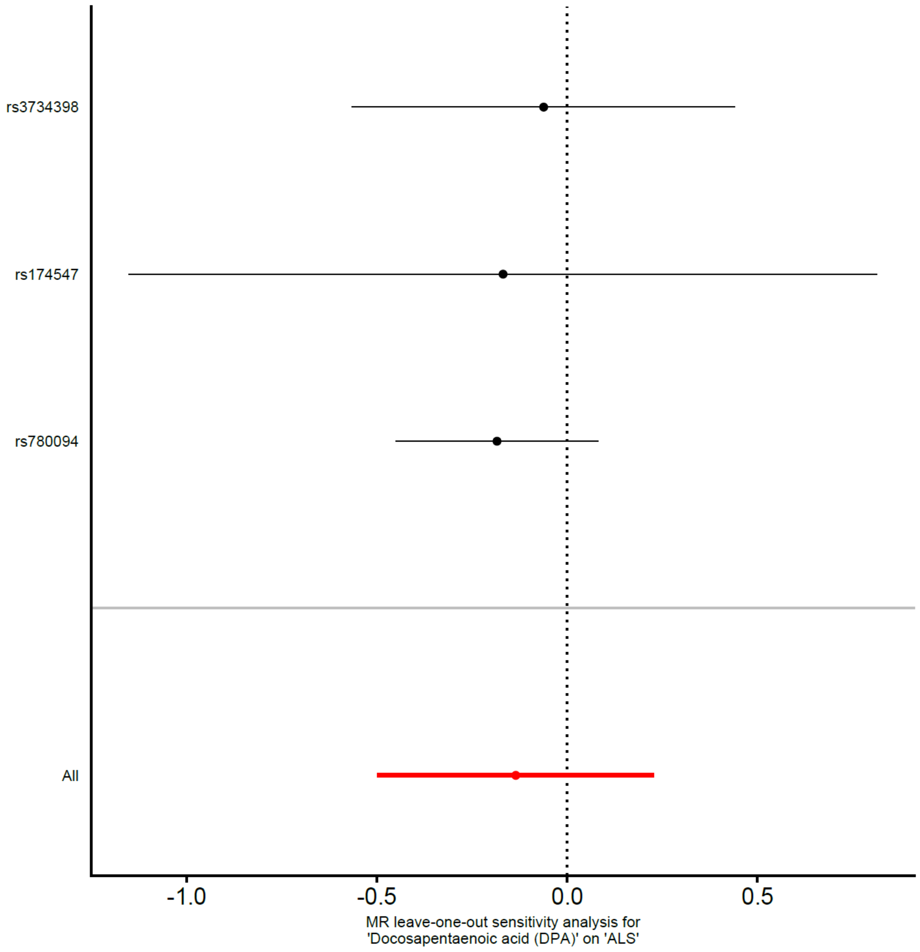


Supplemental Figure S2E. Forrest plot of docosapentaenoic (DPA) and amyotrophic lateral sclerosis

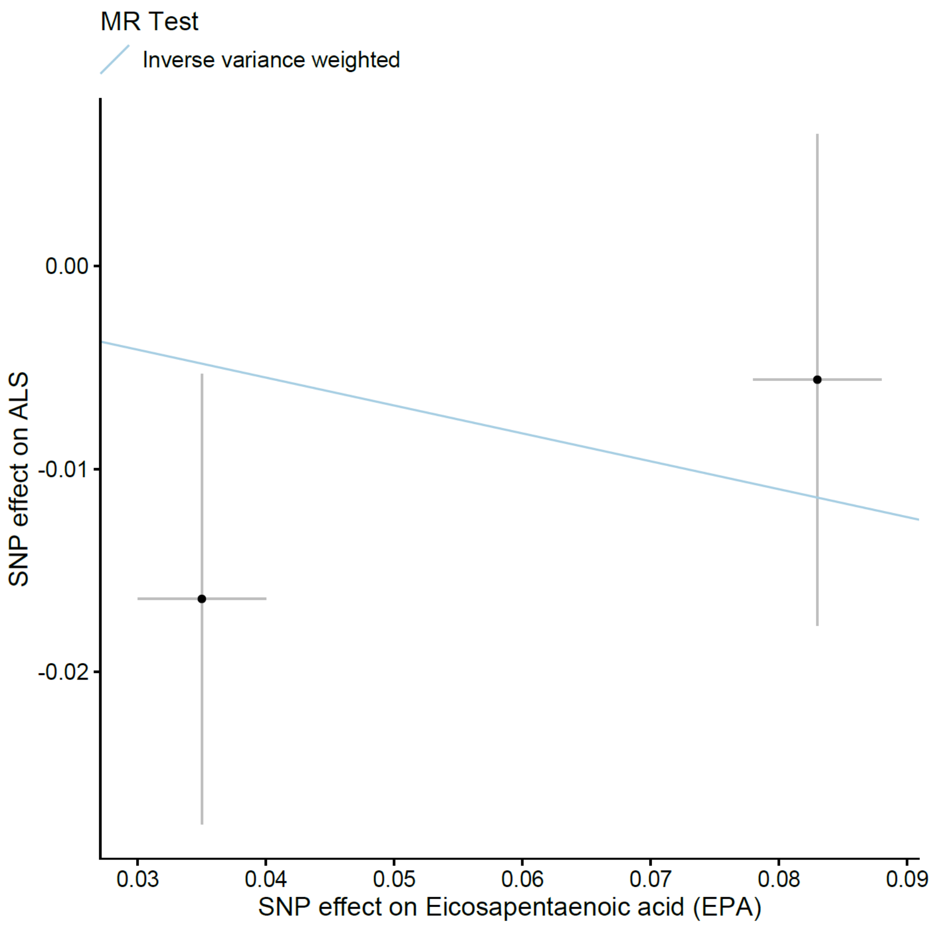


Supplemental Figure S2F. Scatterplot of eicosapentaenoic (EPA) and amyotrophic lateral sclerosis

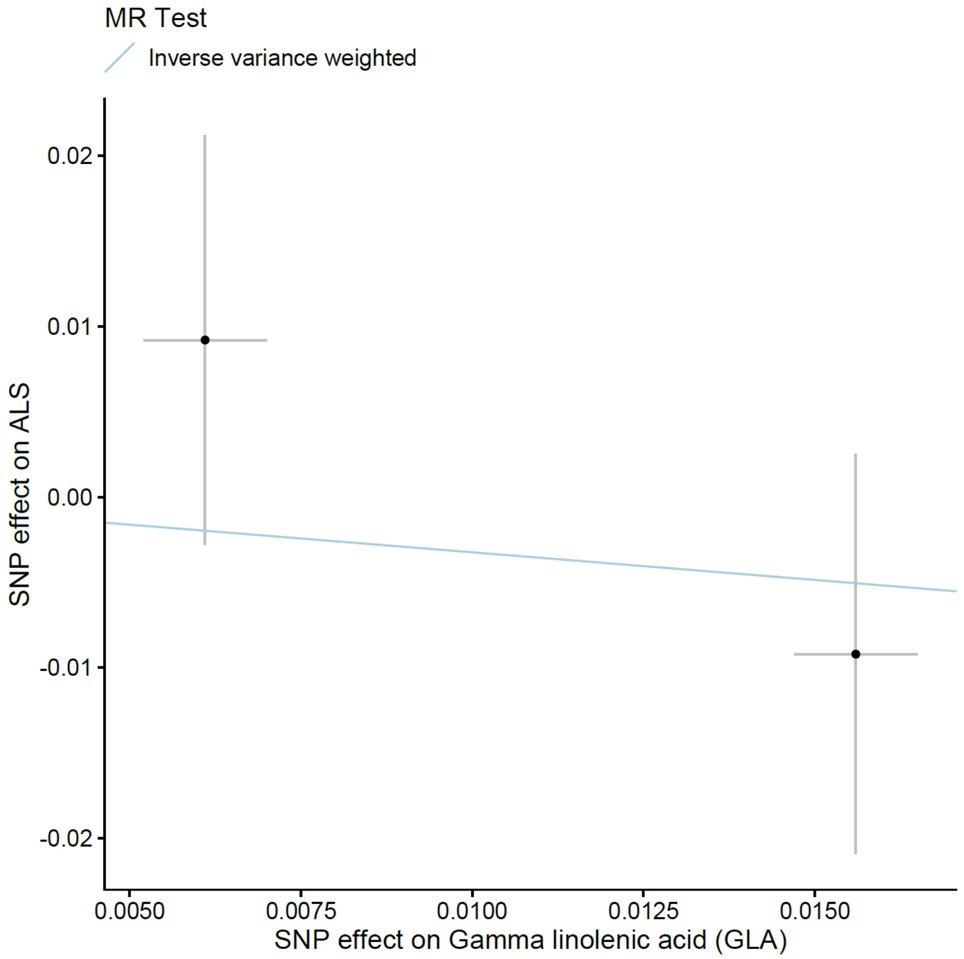


Supplemental Figure S2G. Scatterplot of gamma linolenic acid (GLA) and amyotrophic lateral sclerosis

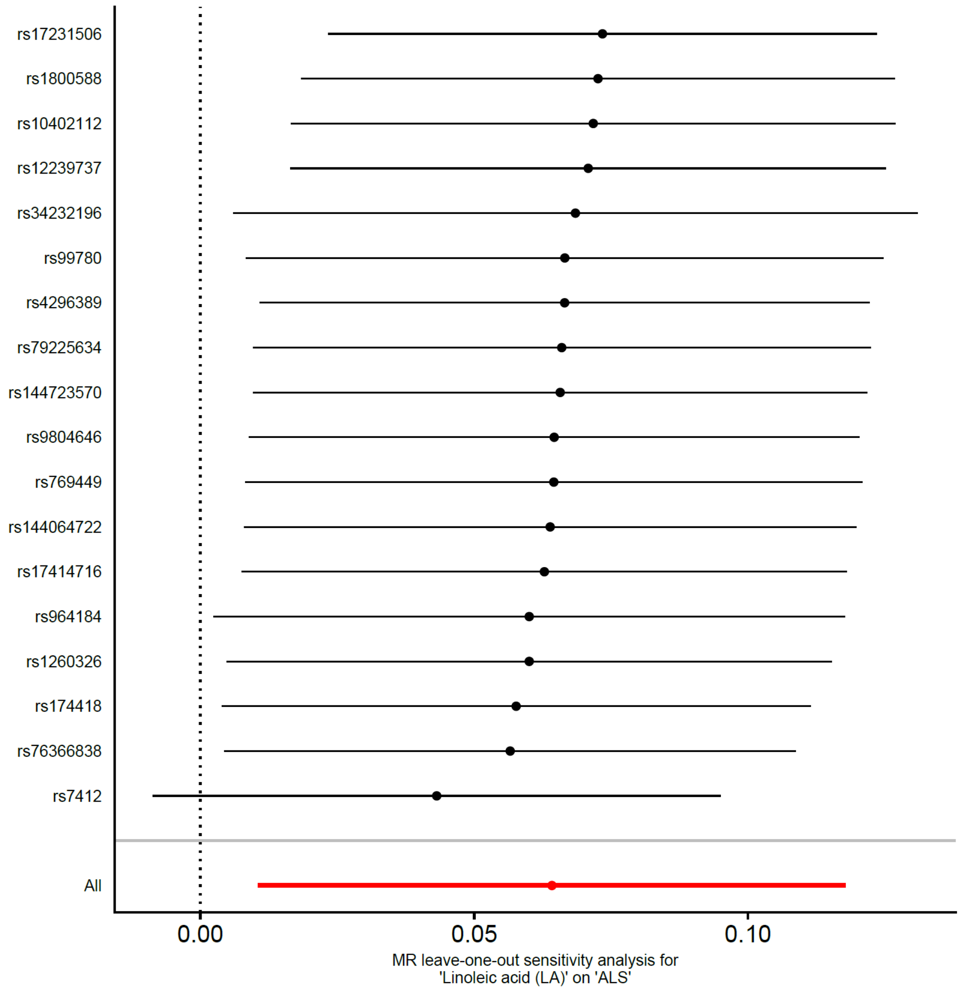


Supplemental Figure S2H. Forrest plot of linoleic acid and amyotrophic lateral sclerosis
