# Supplementary material for: Dietary-Derived Essential Nutrients and Amyotrophic Lateral Sclerosis: A Two-Sample Mendelian Randomization Study
Source: Nutrients. 2022 Feb 22;14(5):920. doi: 10.3390/nu14050920 (PMC8912818; doi:10.3390/nu14050920)
Supplement: Supplementary file 1 [file nutrients-14-00920-s001.zip › supplementary materials/additional file 3 Supplementary Figure 3-4.pdf]

Dietary derived essential nutrients and amyotrophic lateral sclerosis: a two-sample mendelian randomization study

Kailin Xia[1,2,3#], Yajun Wang[1,2,3#], Linjing Zhang[1,2,3], Lu Tang[1,2,3], Gan Zhang[1,2,3], Tao Huang[4], Ninghao Huang[4], Dongsheng Fan[1,2,3*]

[1]Department of Neurology, Peking University Third Hospital, Beijing, China.

[2]Beijing Key Laboratory of Biomarker and Translational Research in Neurodegenerative Diseases, Beijing, China

[3]Key Laboratory for Neuroscience, National Health Commission/Ministry of Education, Peking University, Beijing, China

[4]Department of Epidemiology and Biostatistics, School of Public Health, Peking University, Beijing, China

#KX and YW contributed equally to this article.

*Corresponding author: Dr. Dongsheng Fan,

Department of Neurology, Peking University Third Hospital,

49 North Garden Road, Haidian District, Beijing 100191, People's Republic of China.

E-mail:dsfan2010@aliyun.com

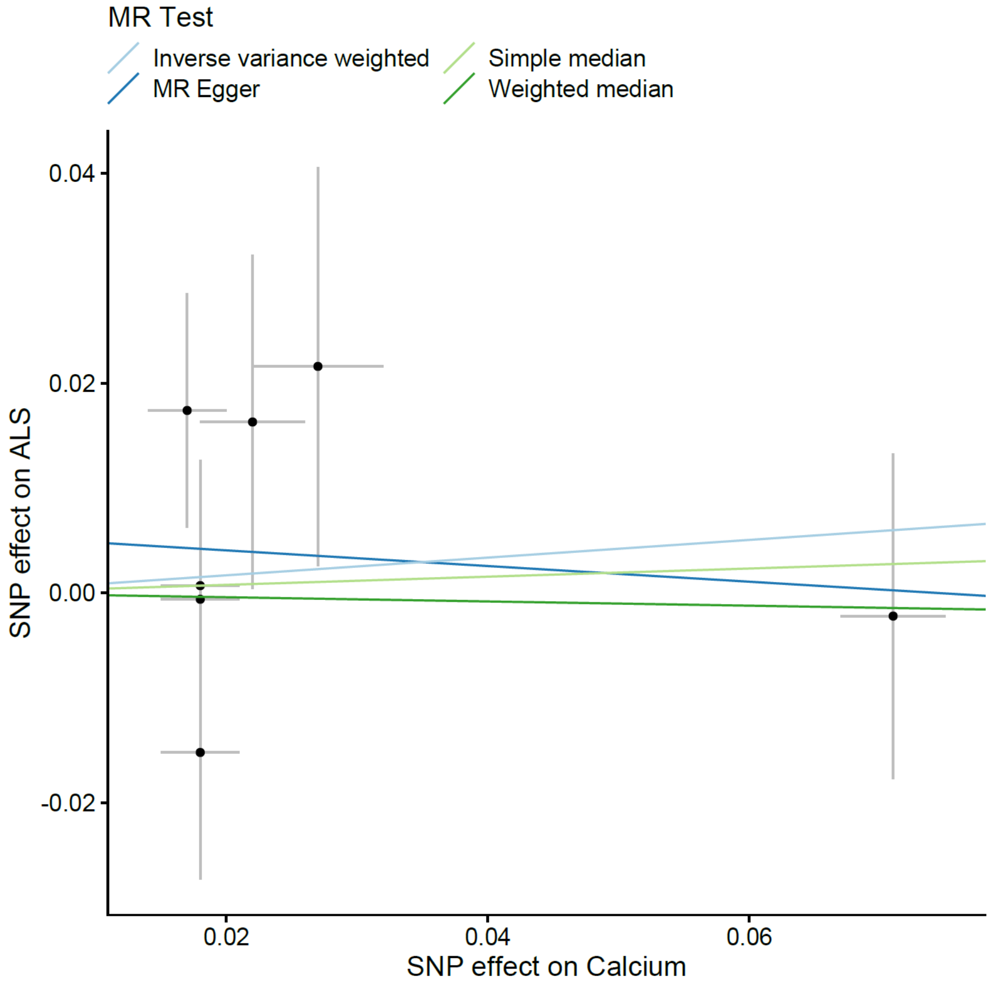

Supplemental Figure S3A. Scatterplot of calcium and amyotrophic lateral sclerosis

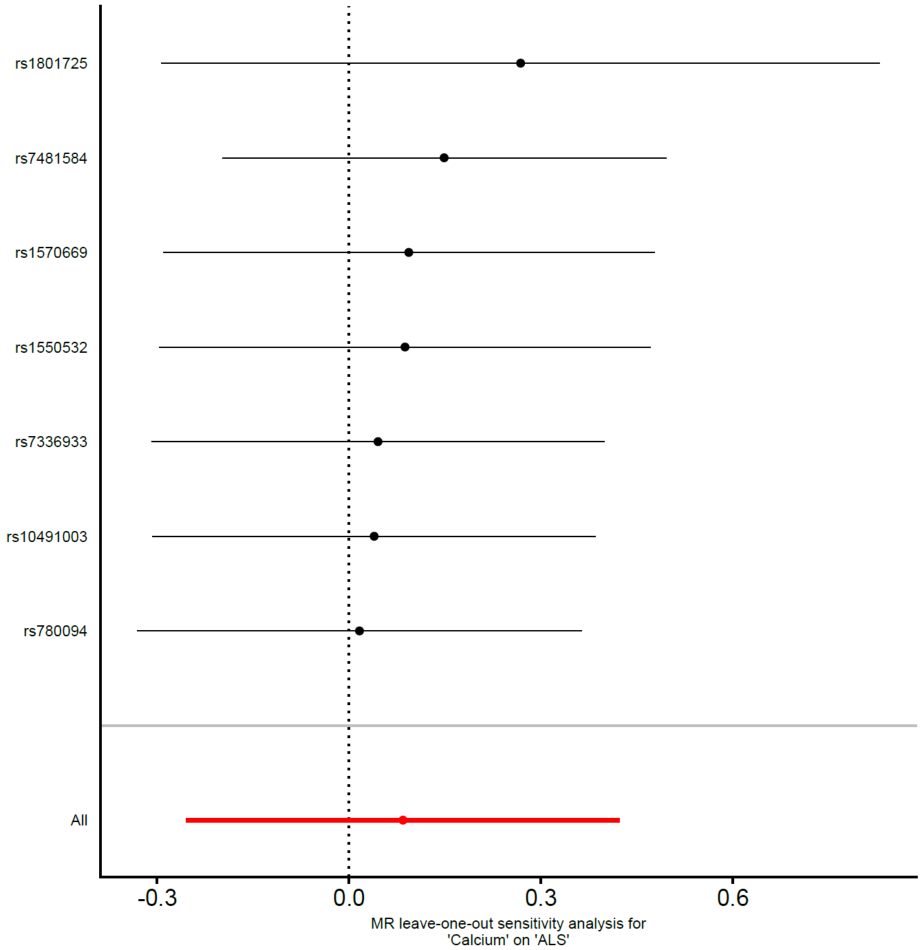

Supplemental Figure S3B. Forrest plot of calcium and amyotrophic lateral sclerosis

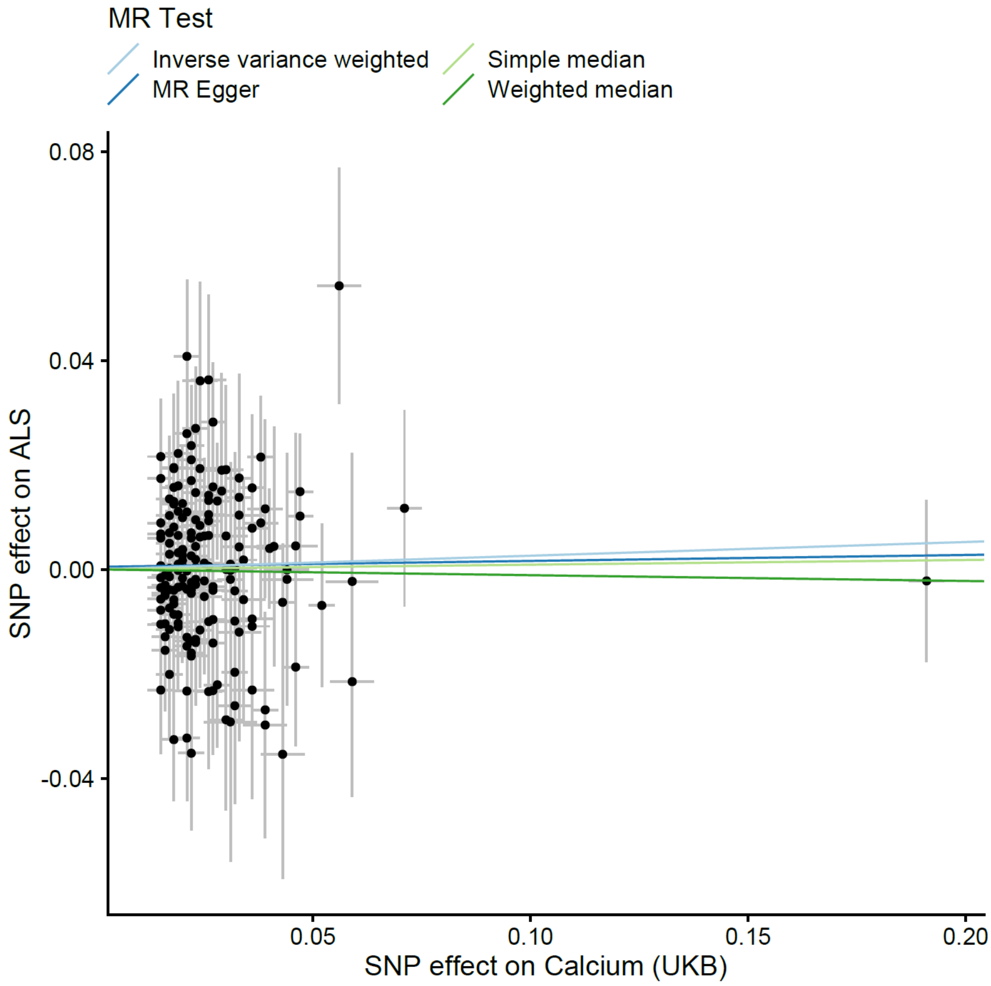

Supplemental Figure S3C. Scatterplot of calcium (UKB) and amyotrophic lateral sclerosis

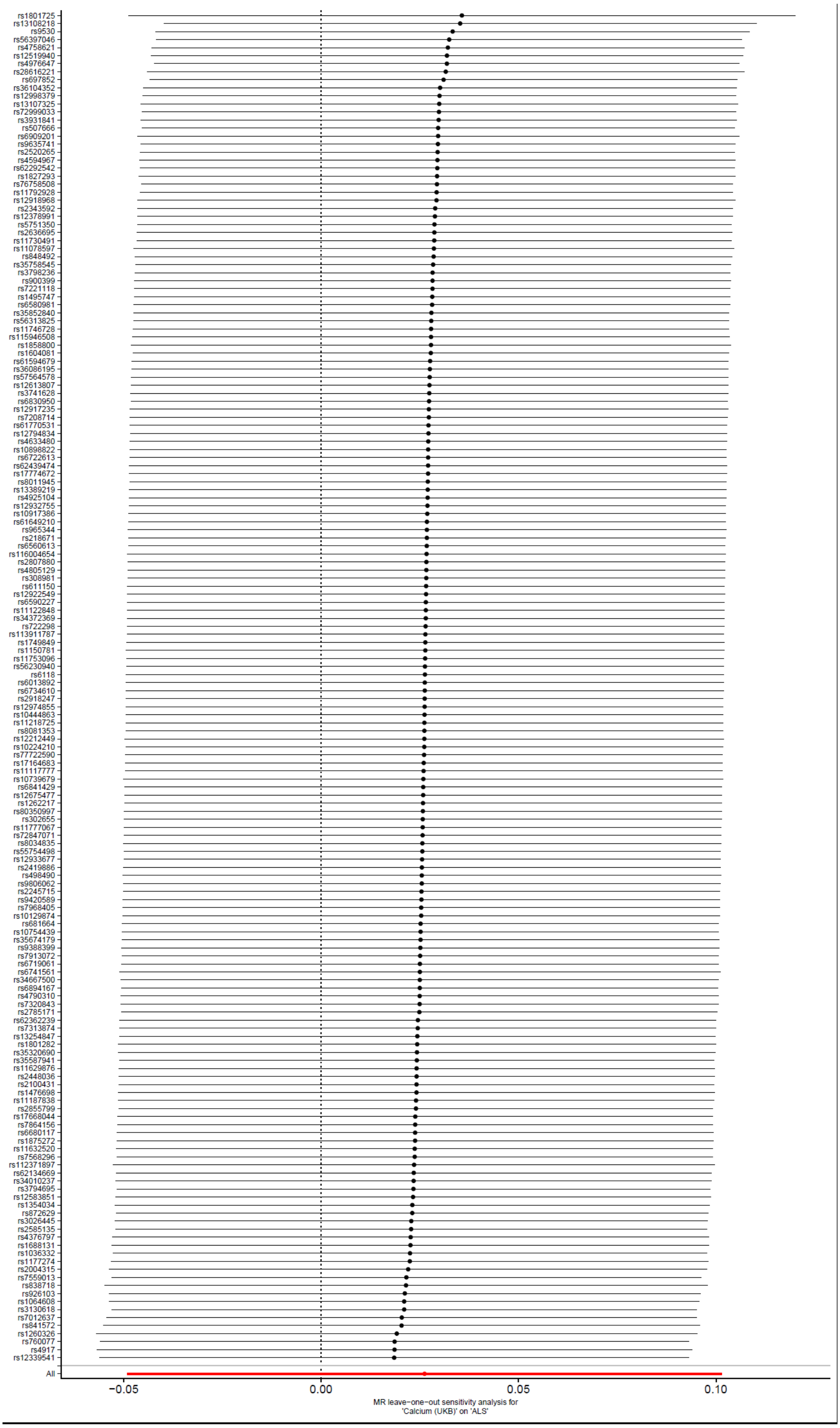

Supplemental Figure S3D. Forrest plot of calcium (UKB) and amyotrophic lateral sclerosis

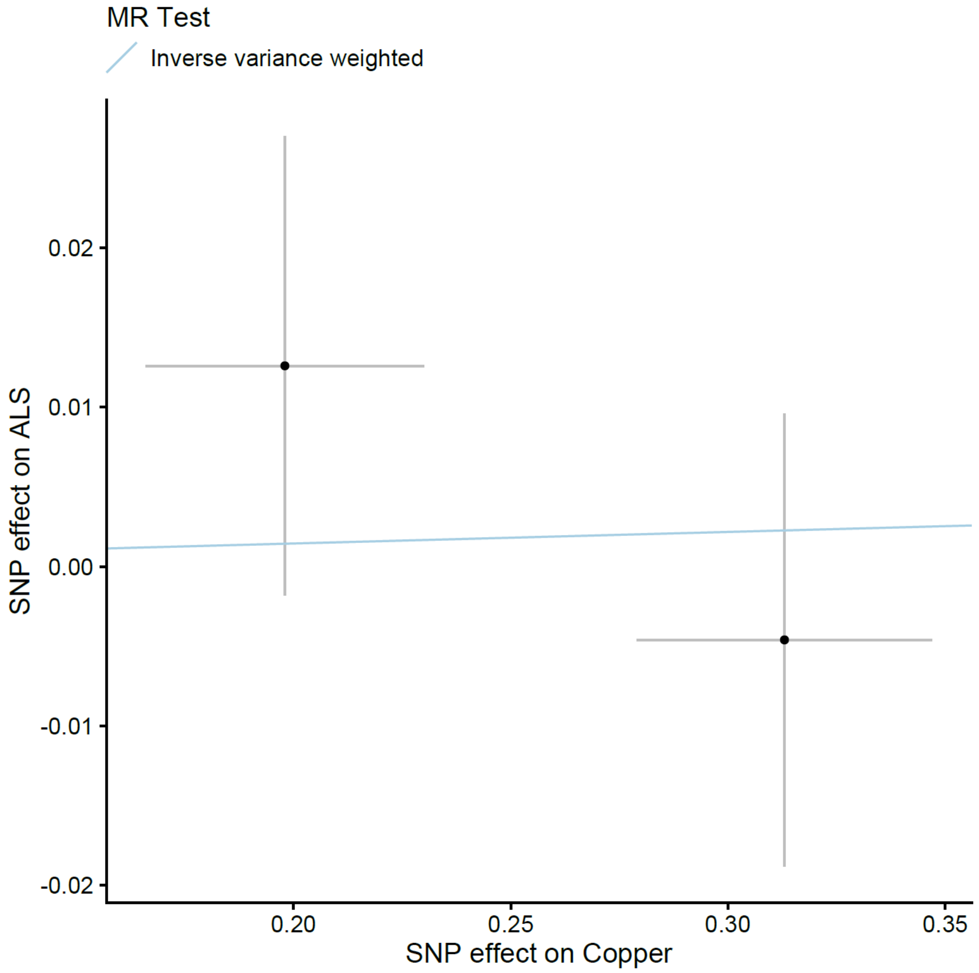

Supplemental Figure S3E. Scatterplot of copper and amyotrophic lateral sclerosis

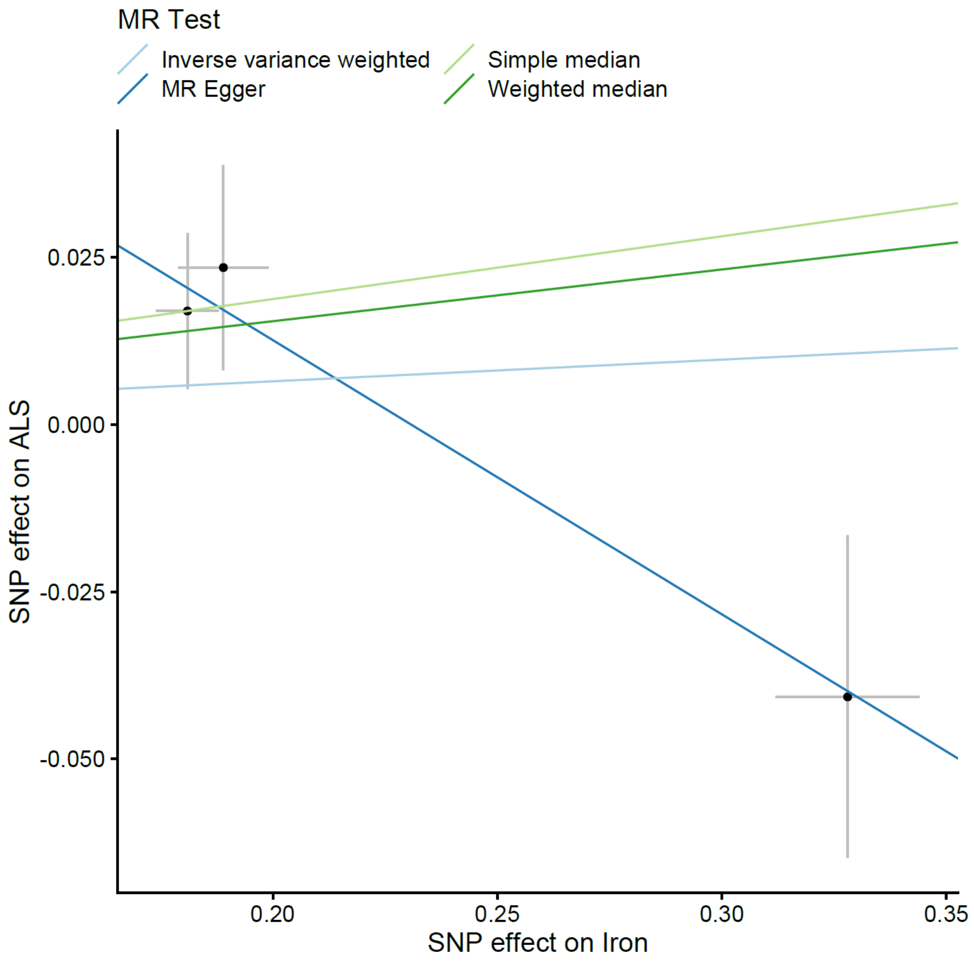

Supplemental Figure S3F. Scatterplot of iron and amyotrophic lateral sclerosis

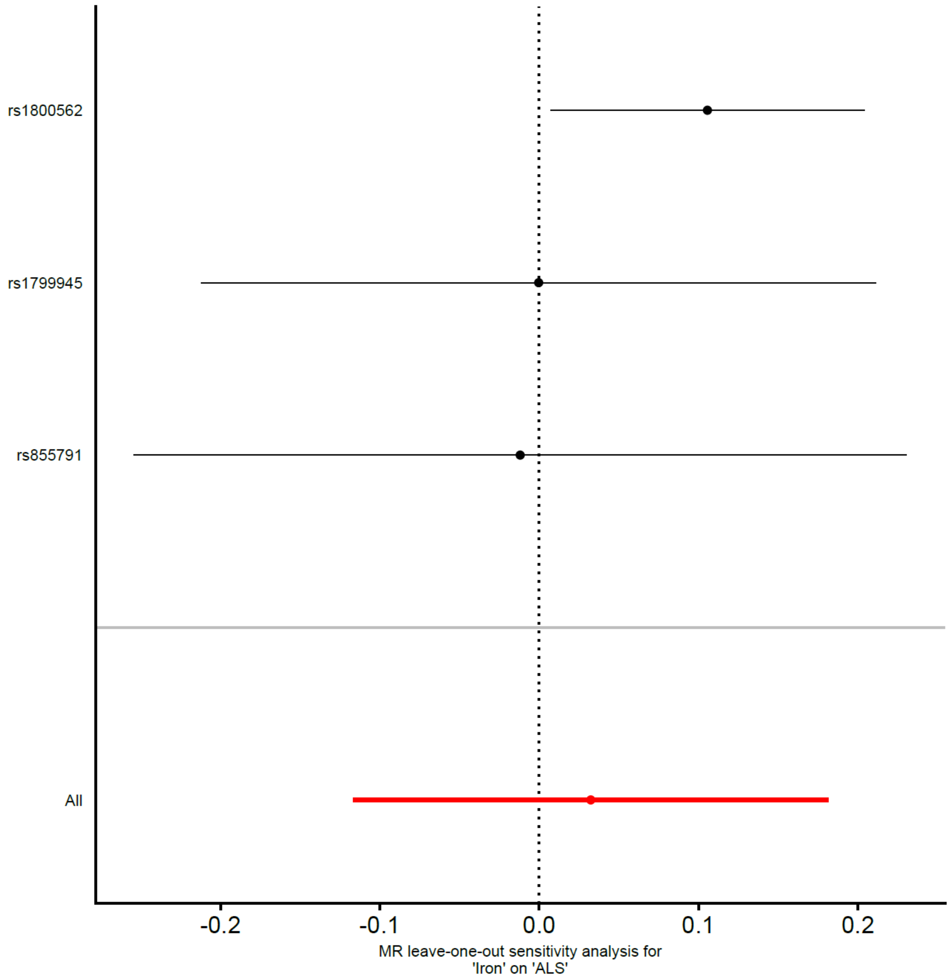

Supplemental Figure S3G. Forrest plot of iron and amyotrophic lateral sclerosis

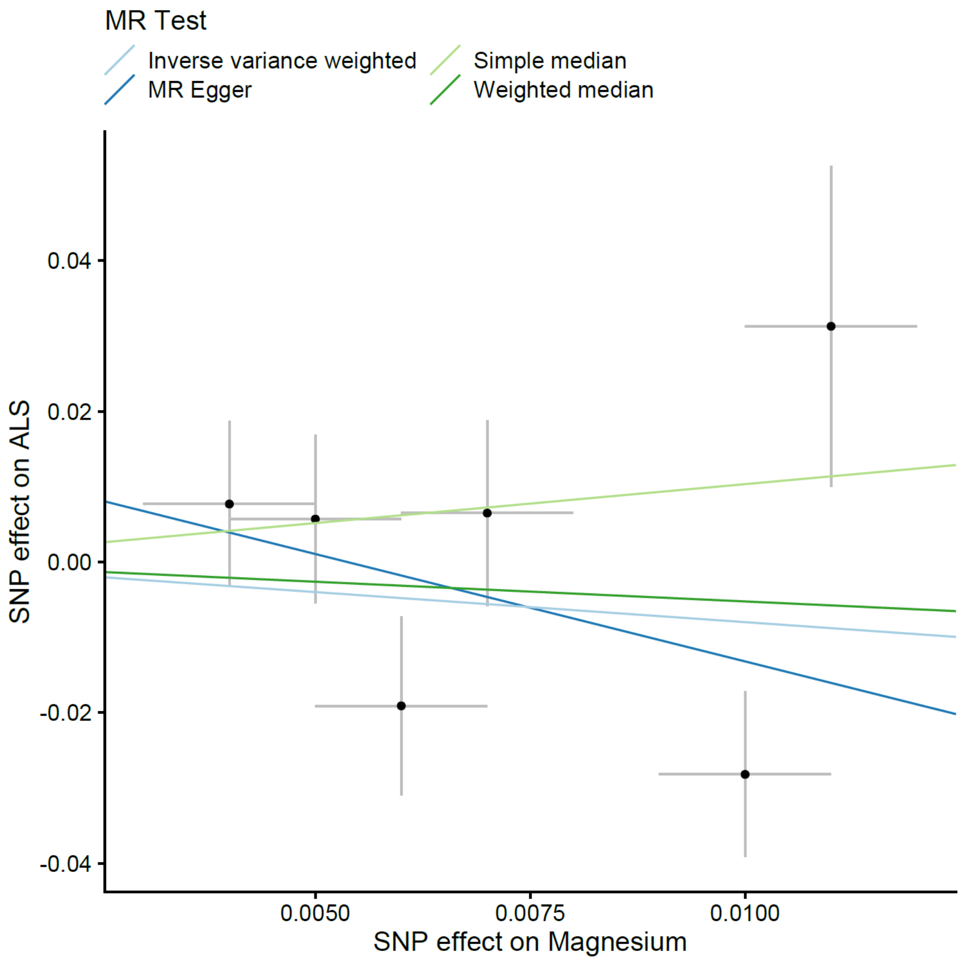

Supplemental Figure S3H. Scatterplot of magnesium and amyotrophic lateral sclerosis

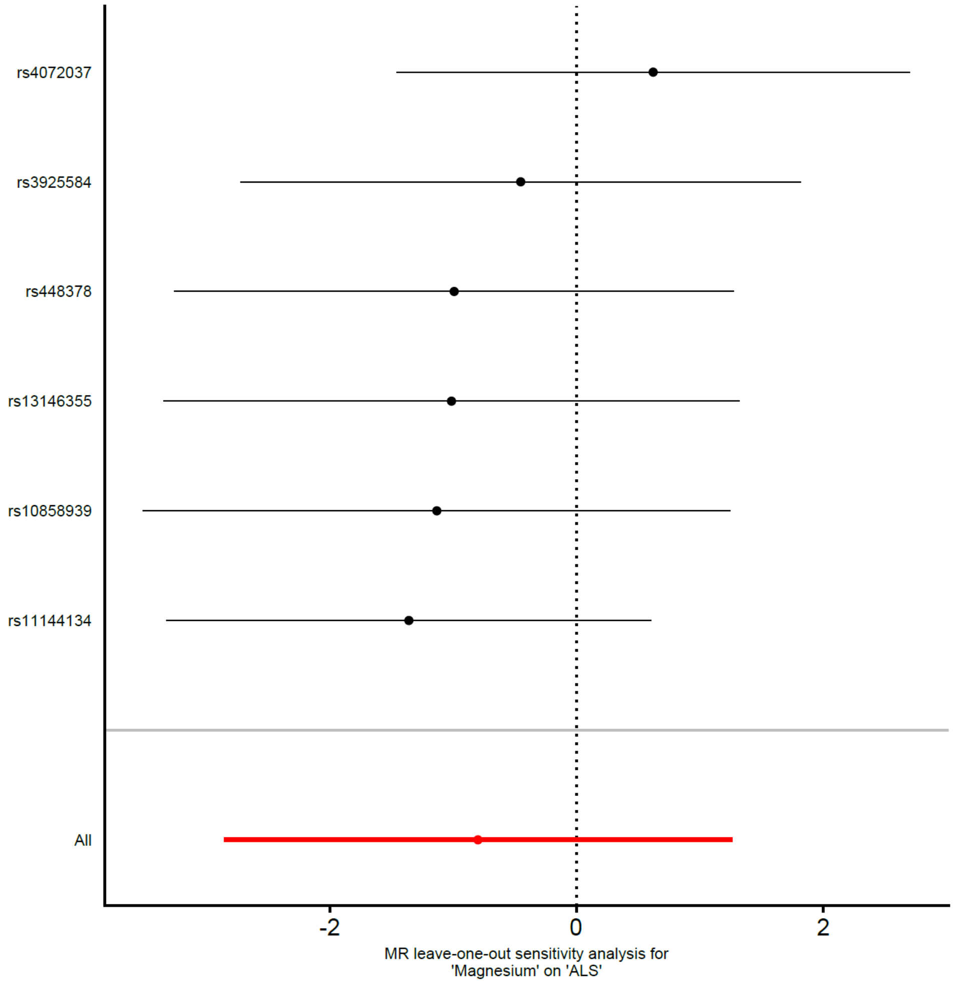

Supplemental Figure S3I. Forrest plot of magnesium and amyotrophic lateral sclerosis

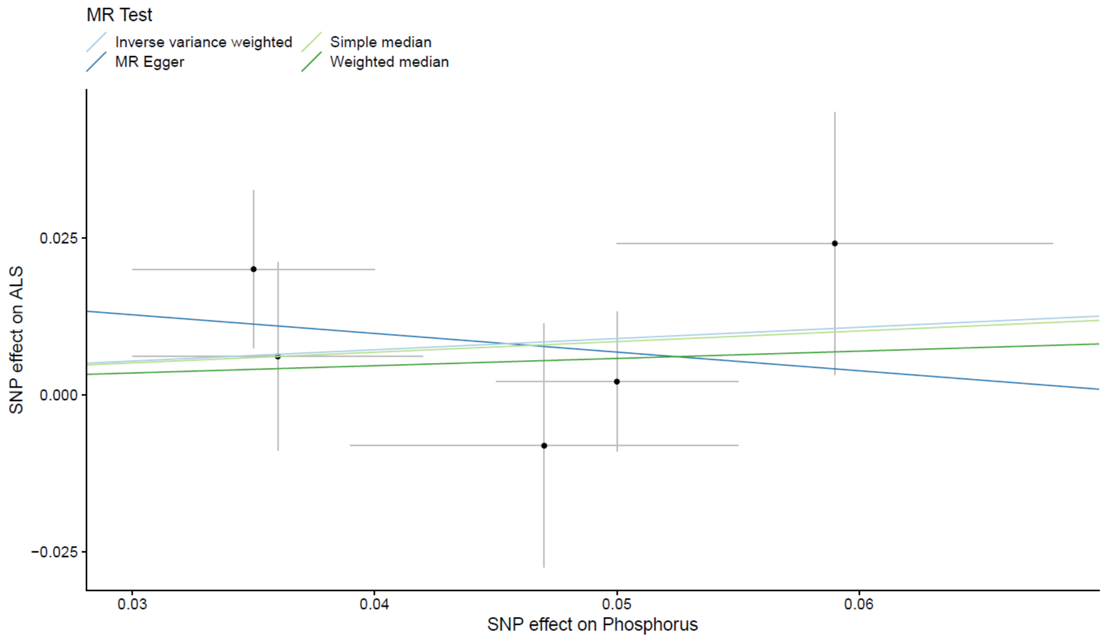

Supplemental Figure S3J. Scatterplot of phosphorus and amyotrophic lateral sclerosis

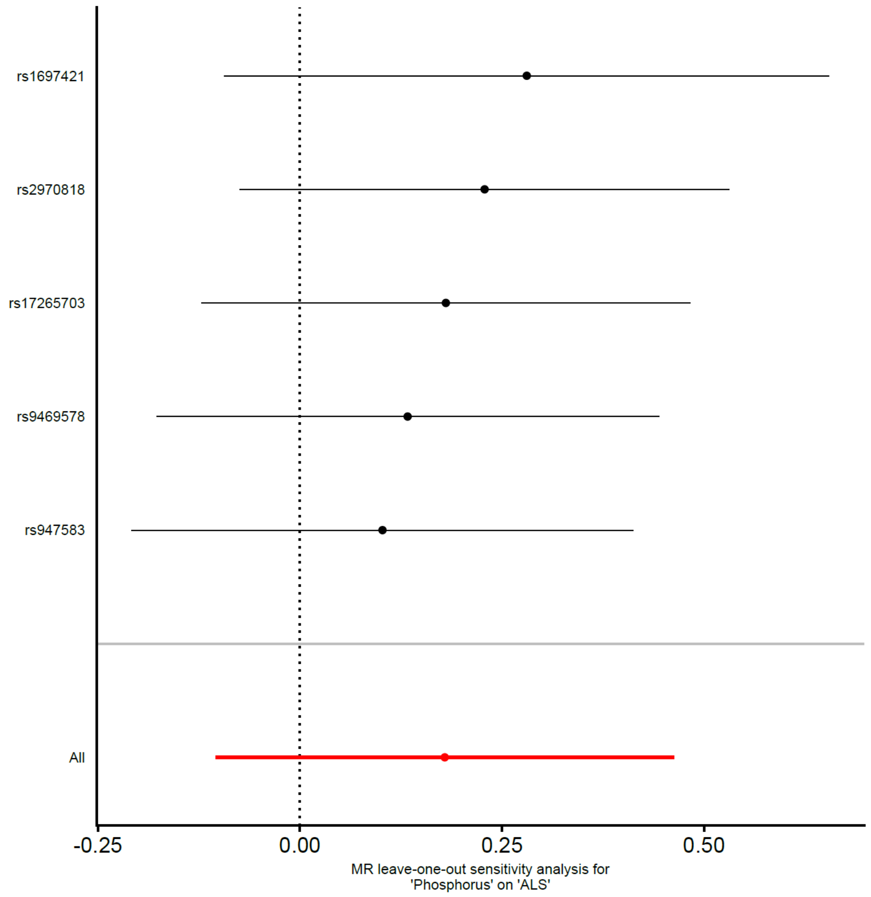

Supplemental Figure S3K. Forrest plot of phosphorus and amyotrophic lateral sclerosis

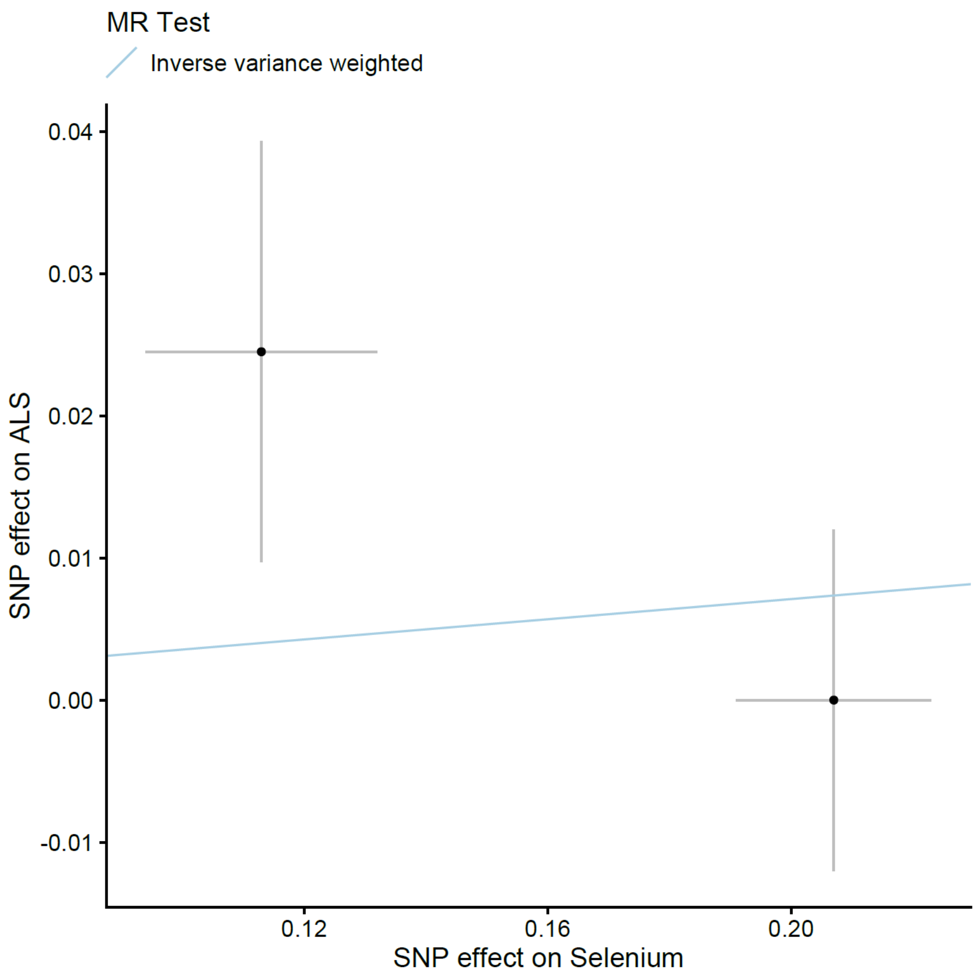

Supplemental Figure S3L. Scatterplot of selenium and amyotrophic lateral sclerosis

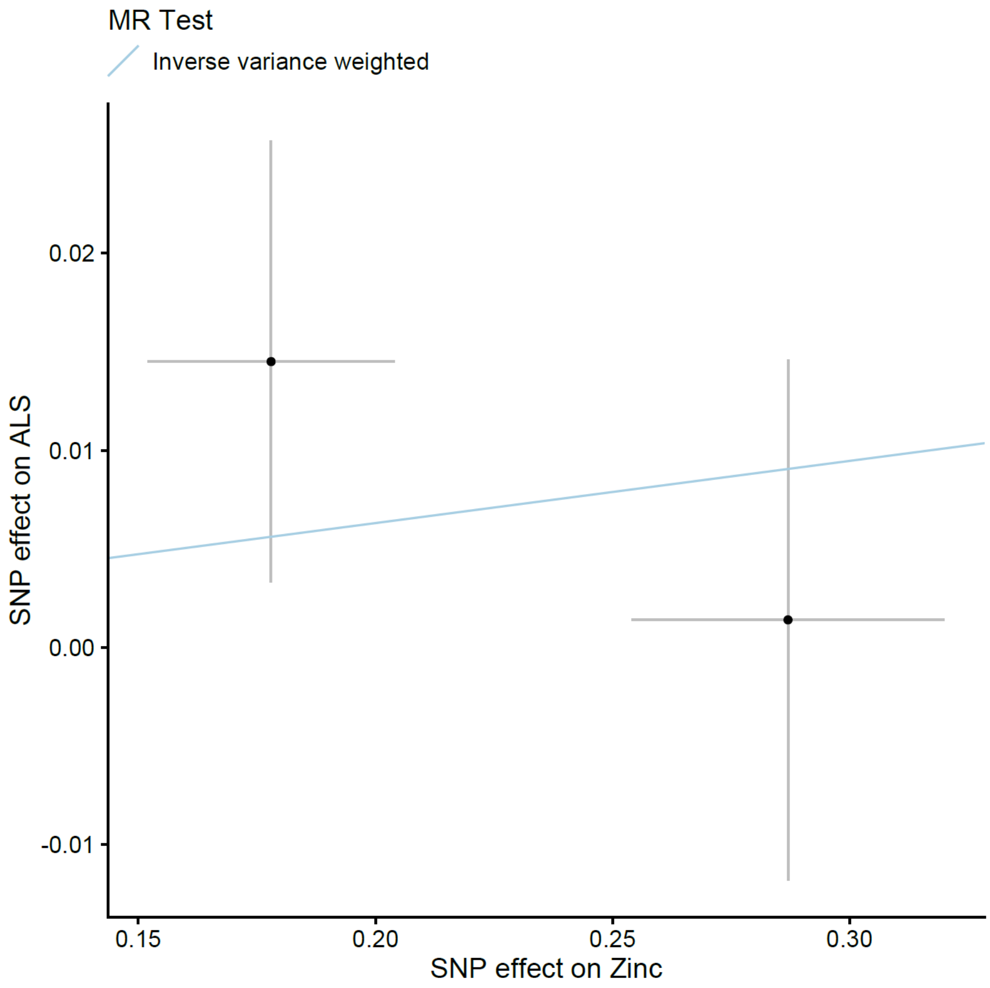

Supplemental Figure S3M. Scatterplot of zinc and amyotrophic lateral sclerosis

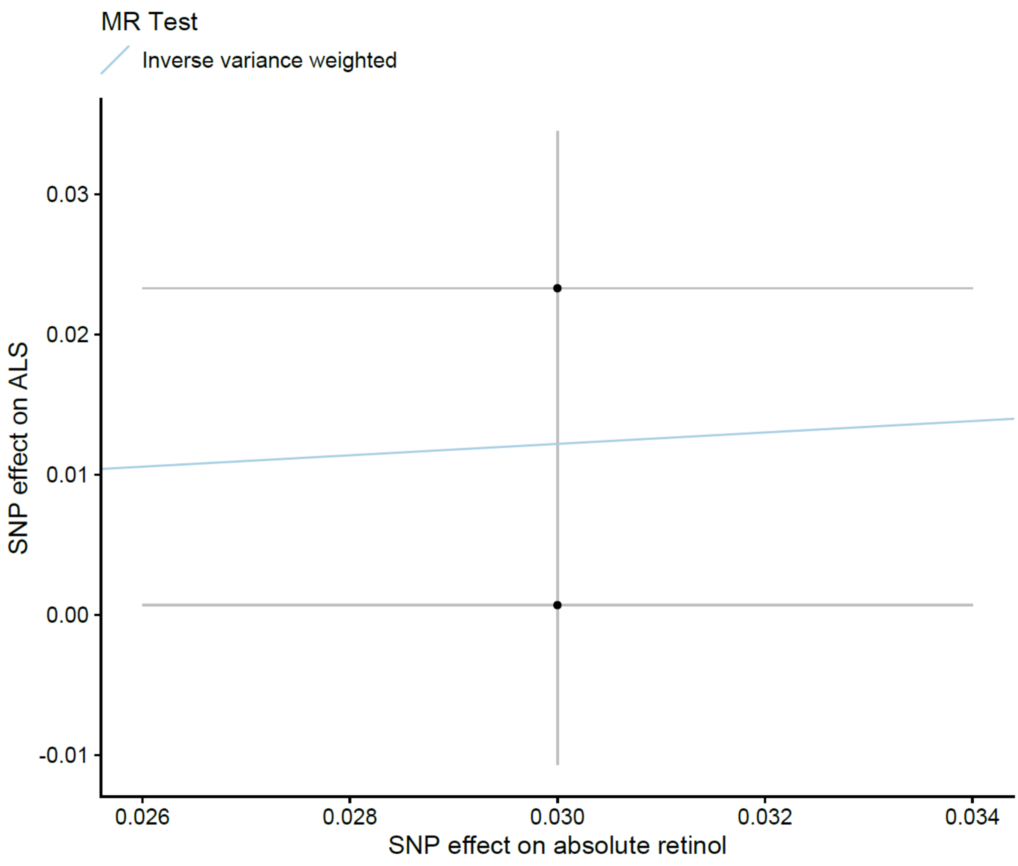

Supplemental Figure S4A. Scatterplot of absolute retinol and amyotrophic lateral sclerosis

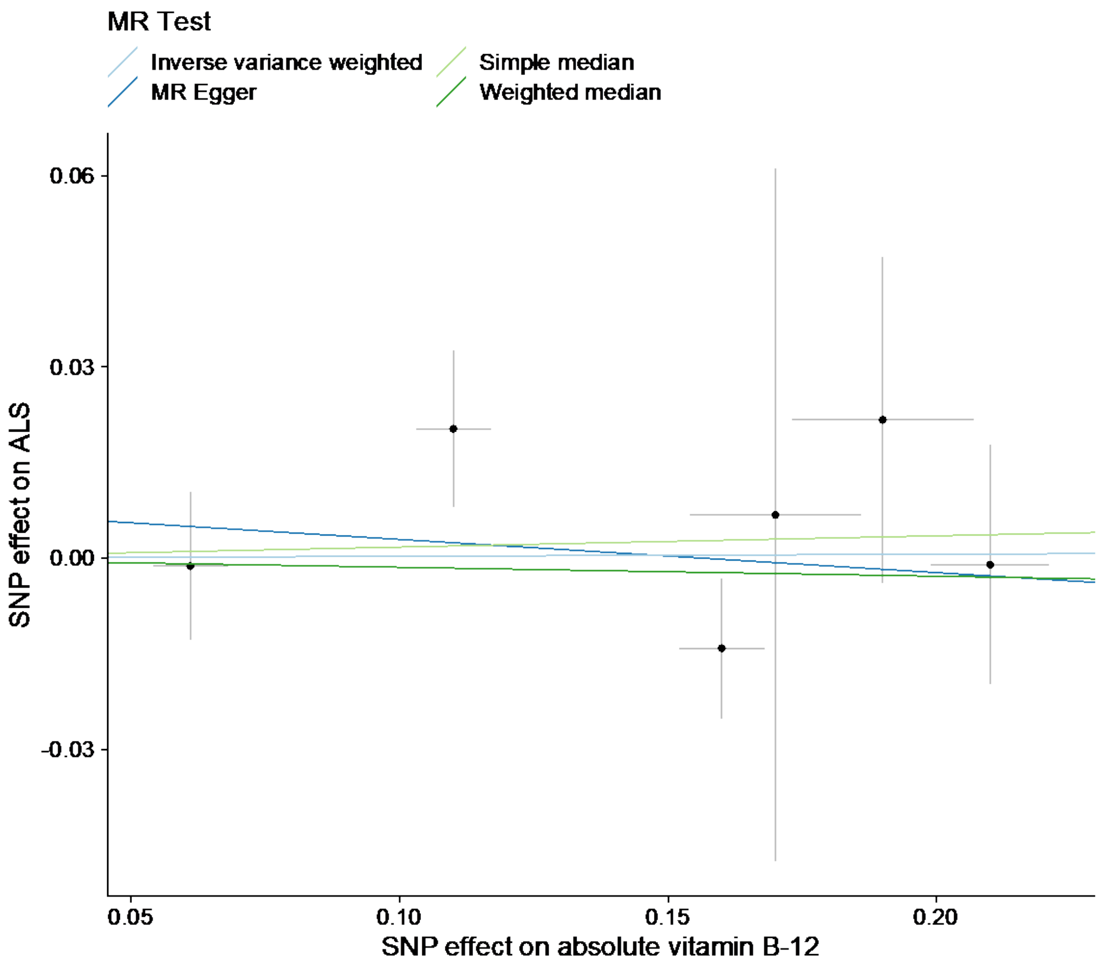

Supplemental Figure S4B. Scatterplot of vitamin B-12 and amyotrophic lateral sclerosis

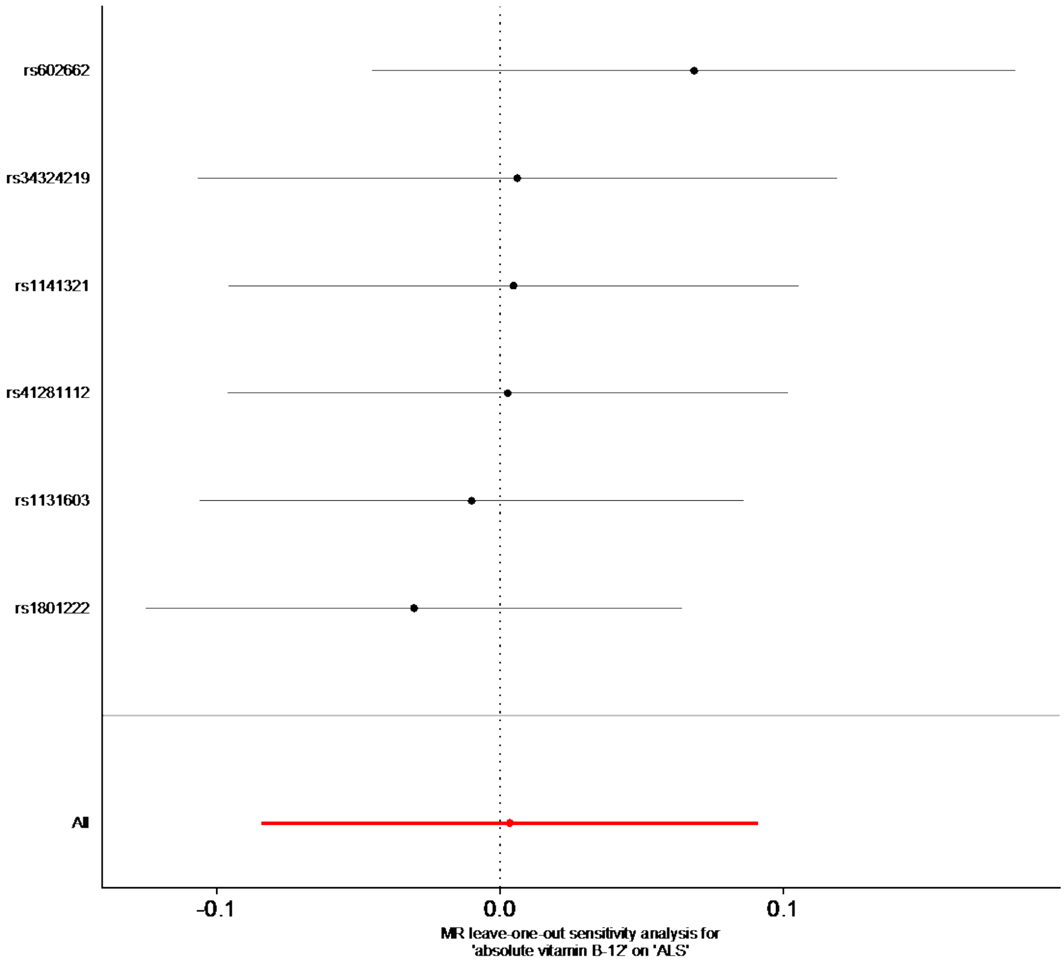

Supplemental Figure S4C. Forrest plot of vitamin B-12 and amyotrophic lateral sclerosis

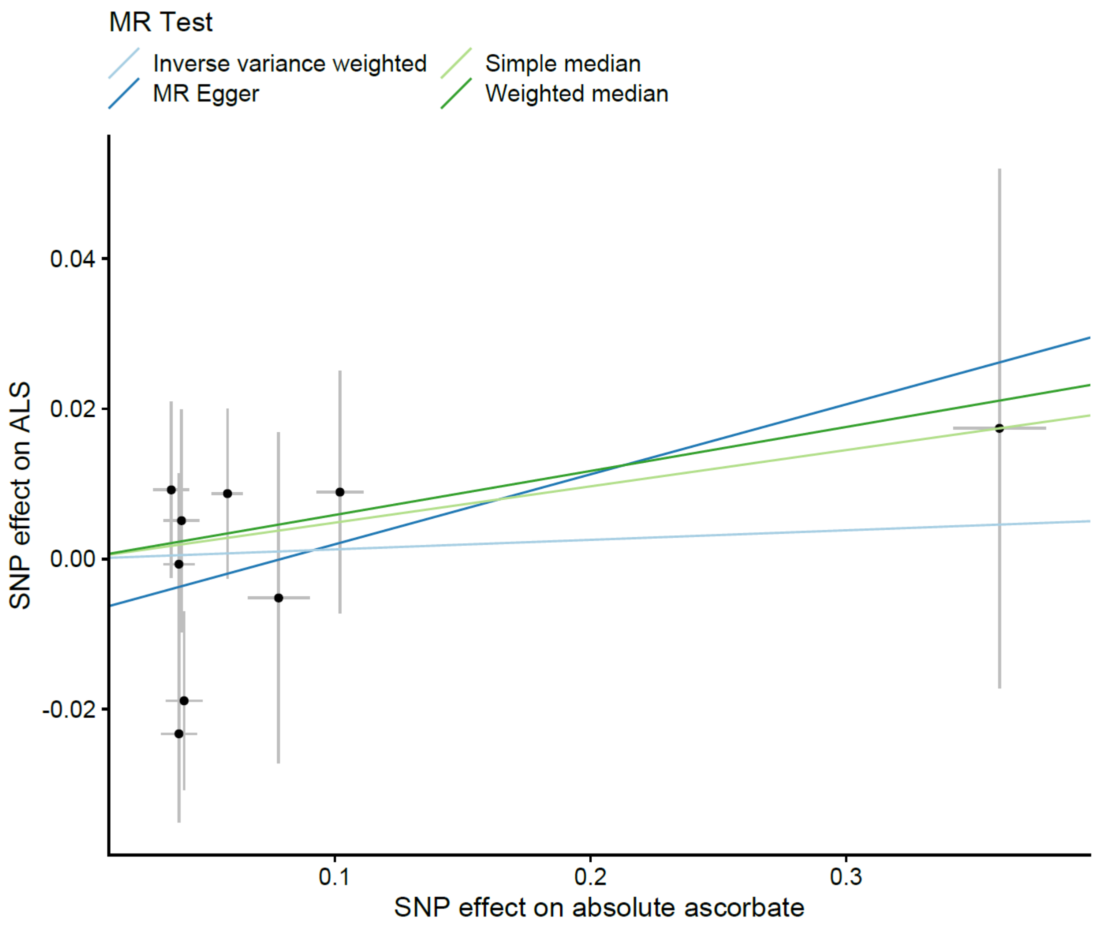

Supplemental Figure S4D. Scatterplot of absolute ascorbate and amyotrophic lateral sclerosis

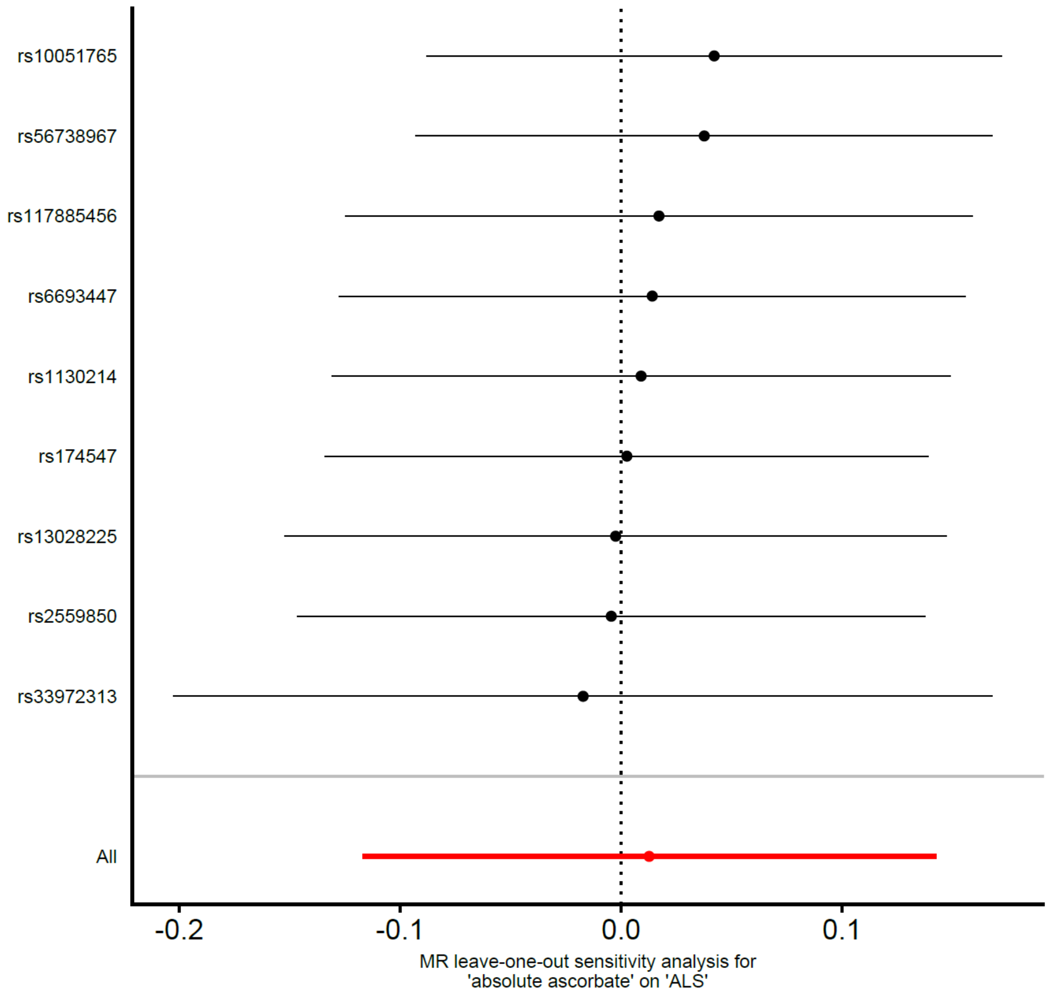

Supplemental Figure S4E. Forrest plot of absolute ascorbate and amyotrophic lateral sclerosis

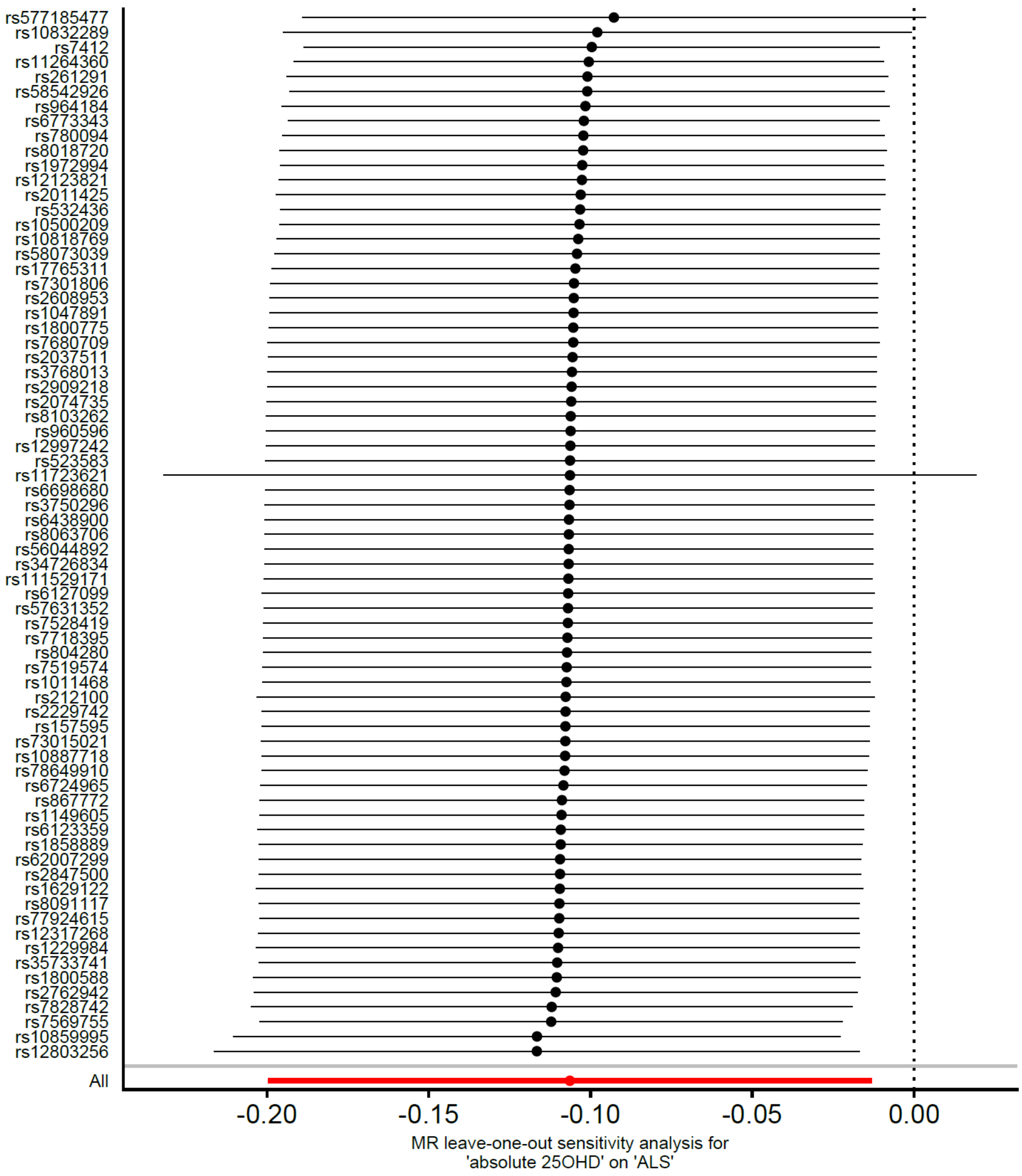

Supplemental Figure S4F. Forrest plot of absolute 25OHD and amyotrophic lateral sclerosis

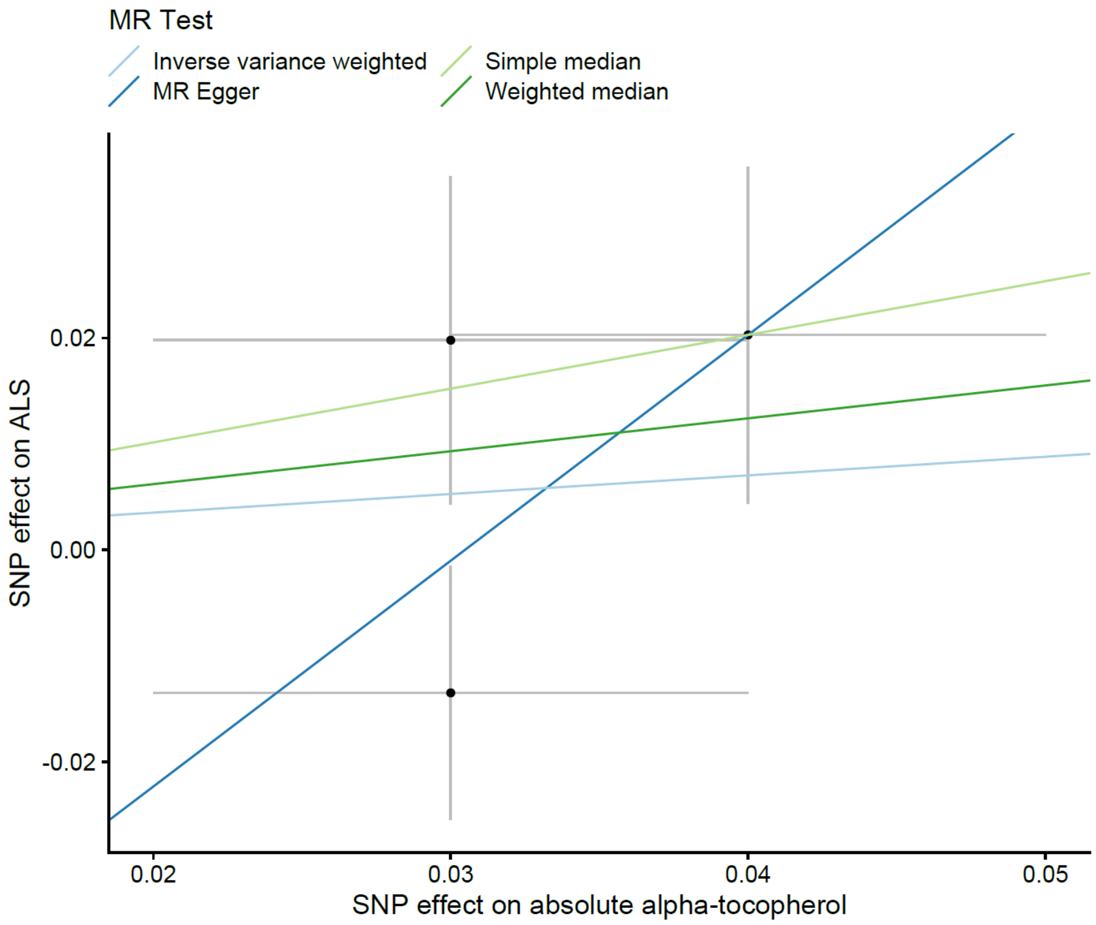

Supplemental Figure S4G. Scatterplot of absolute alpha-tocopherol and amyotrophic lateral sclerosis

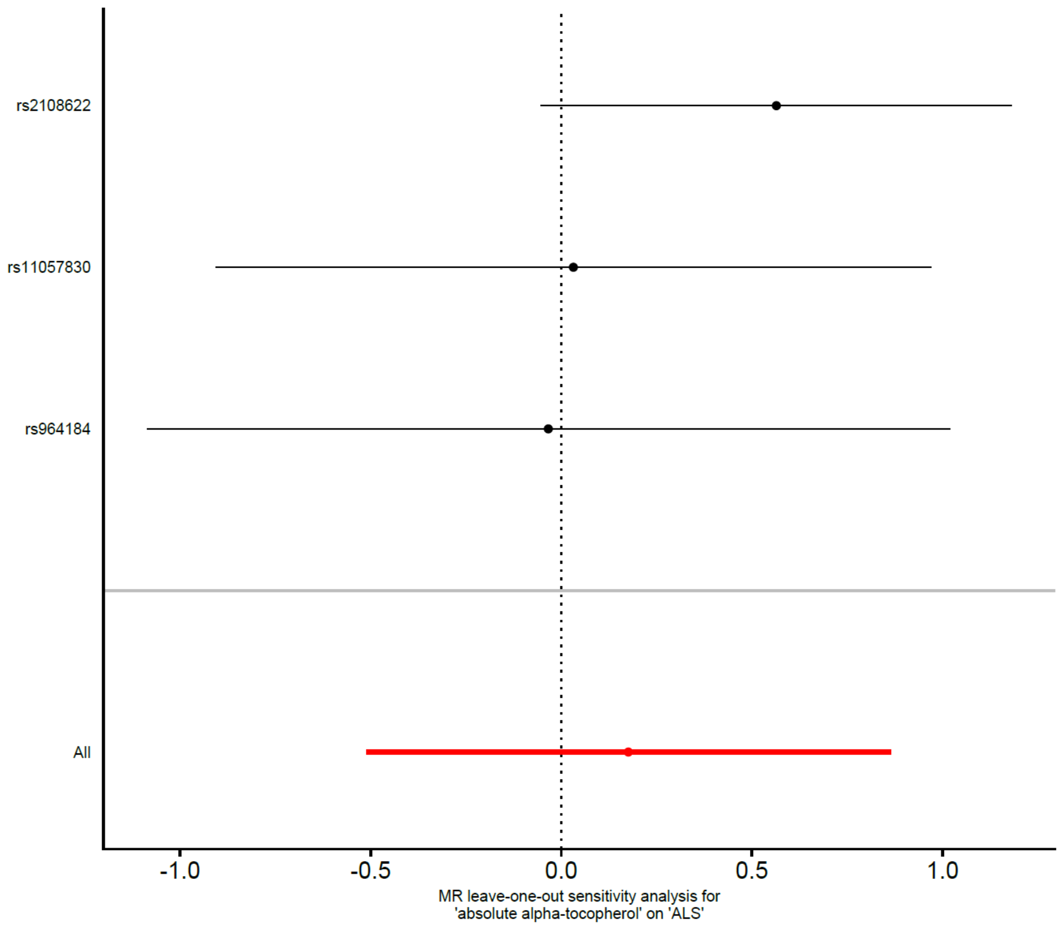

Supplemental Figure S4H. Forrest plot of absolute alpha-tocopherol and amyotrophic lateral sclerosis

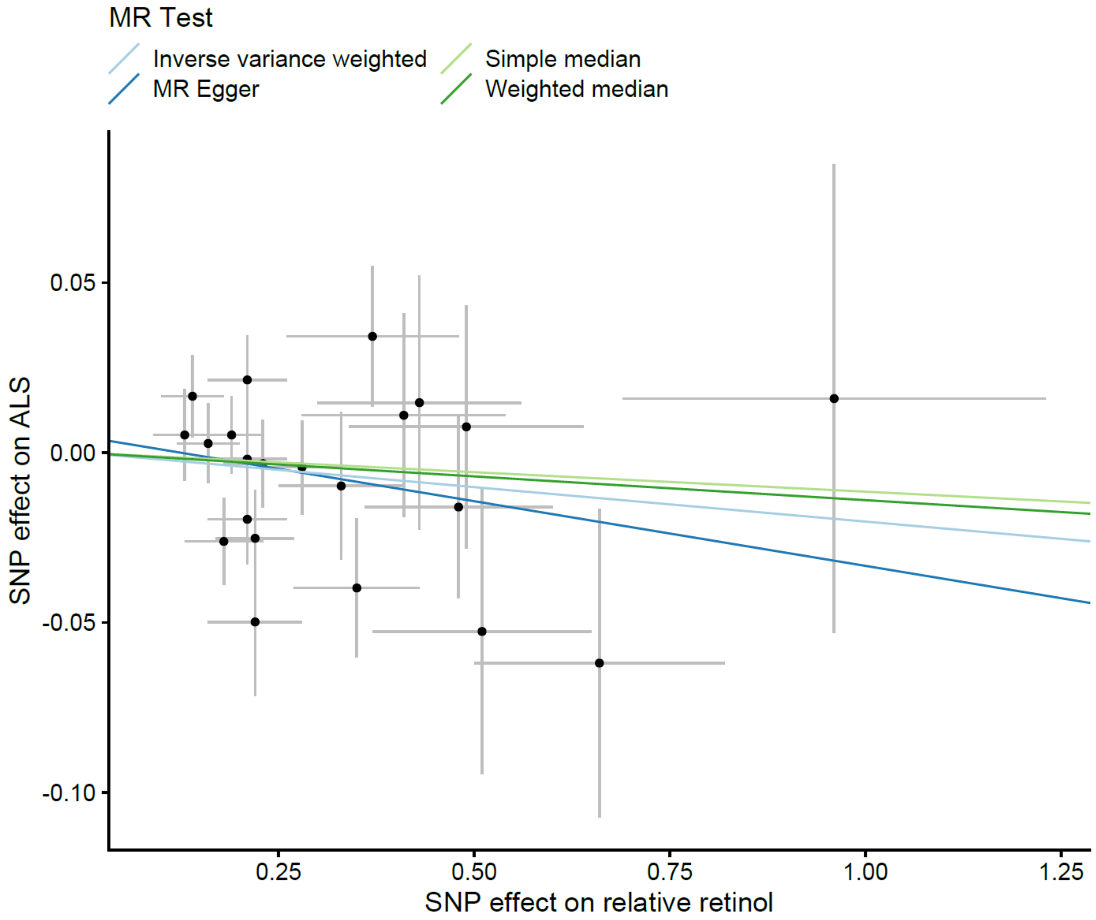

Supplemental Figure S4I. Scatterplot of relative retinol and amyotrophic lateral sclerosis

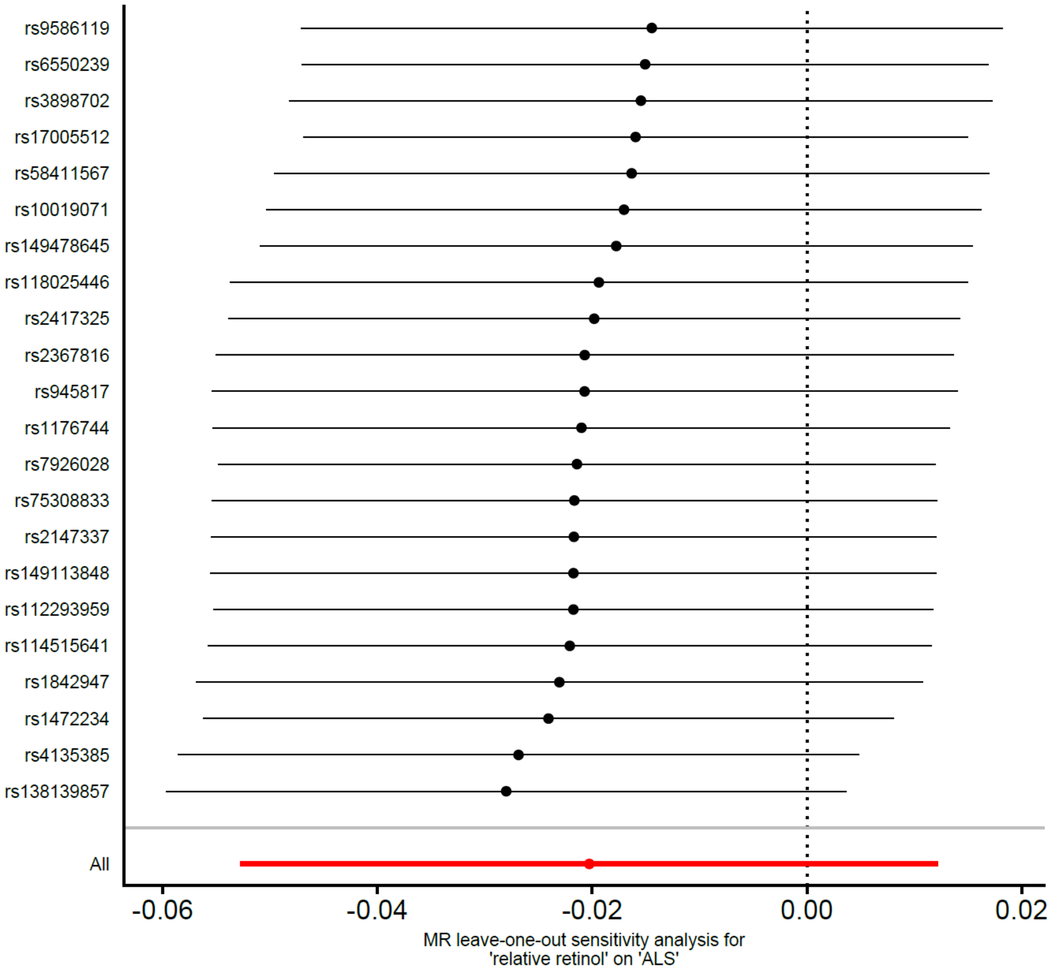

Supplemental Figure S4J. Forrest plot of relative retinol and amyotrophic lateral sclerosis

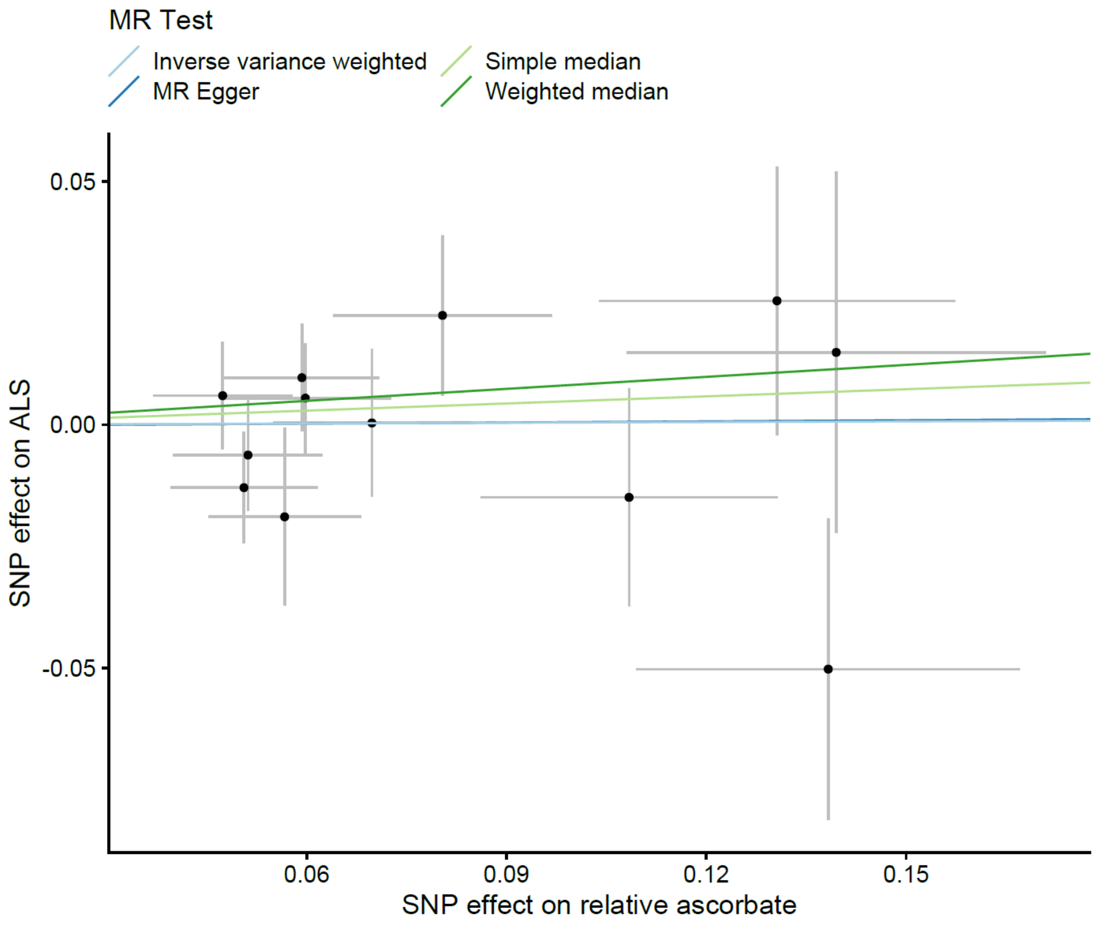

Supplemental Figure S4K. Scatterplot of relative ascorbate and amyotrophic lateral sclerosis

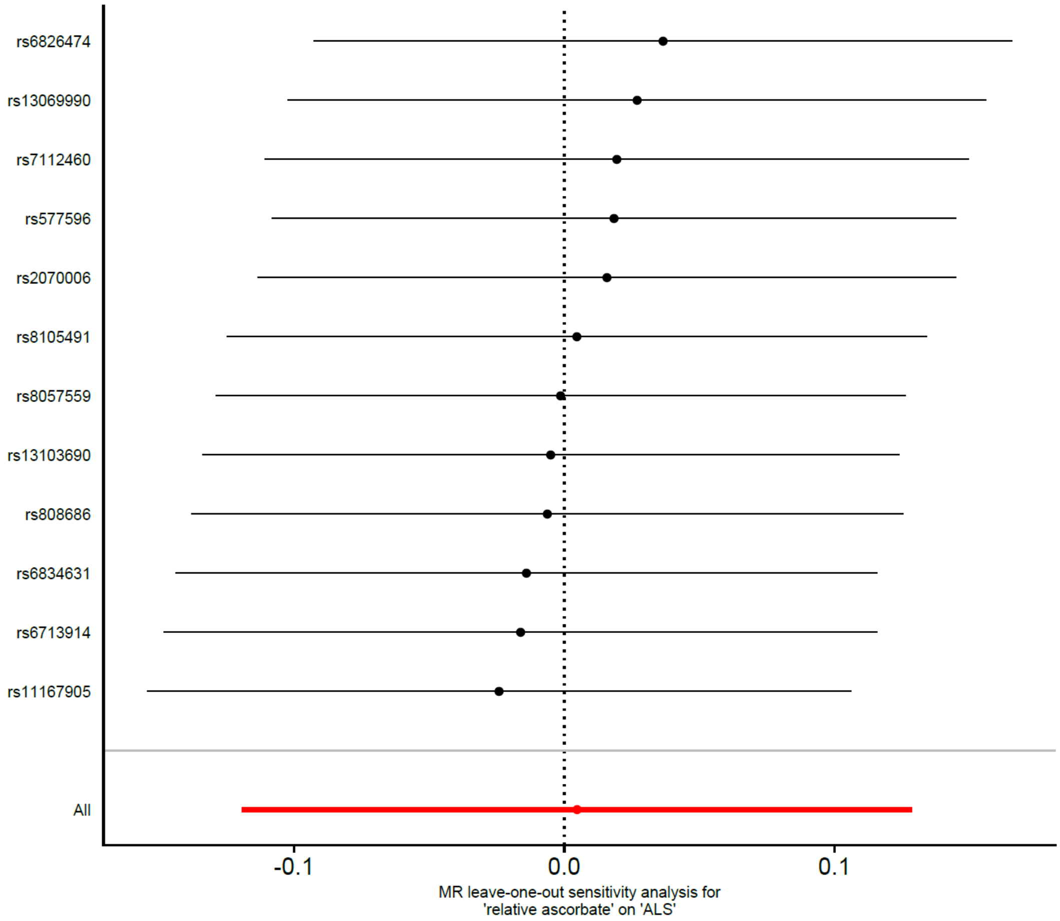

Supplemental Figure S4L. Forrest plot of relative ascorbate and amyotrophic lateral sclerosis

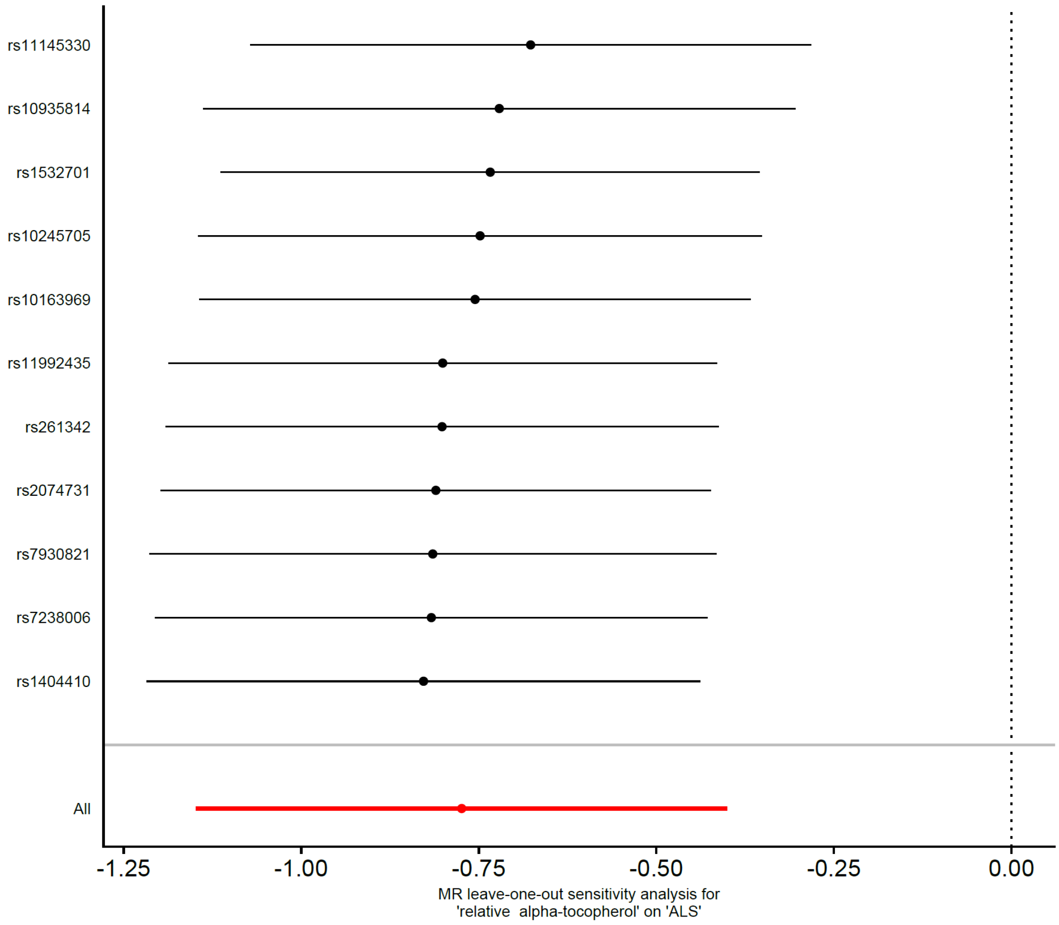

Supplemental Figure S4M. Forrest plot of relative alpha-tocopherol and amyotrophic lateral sclerosis

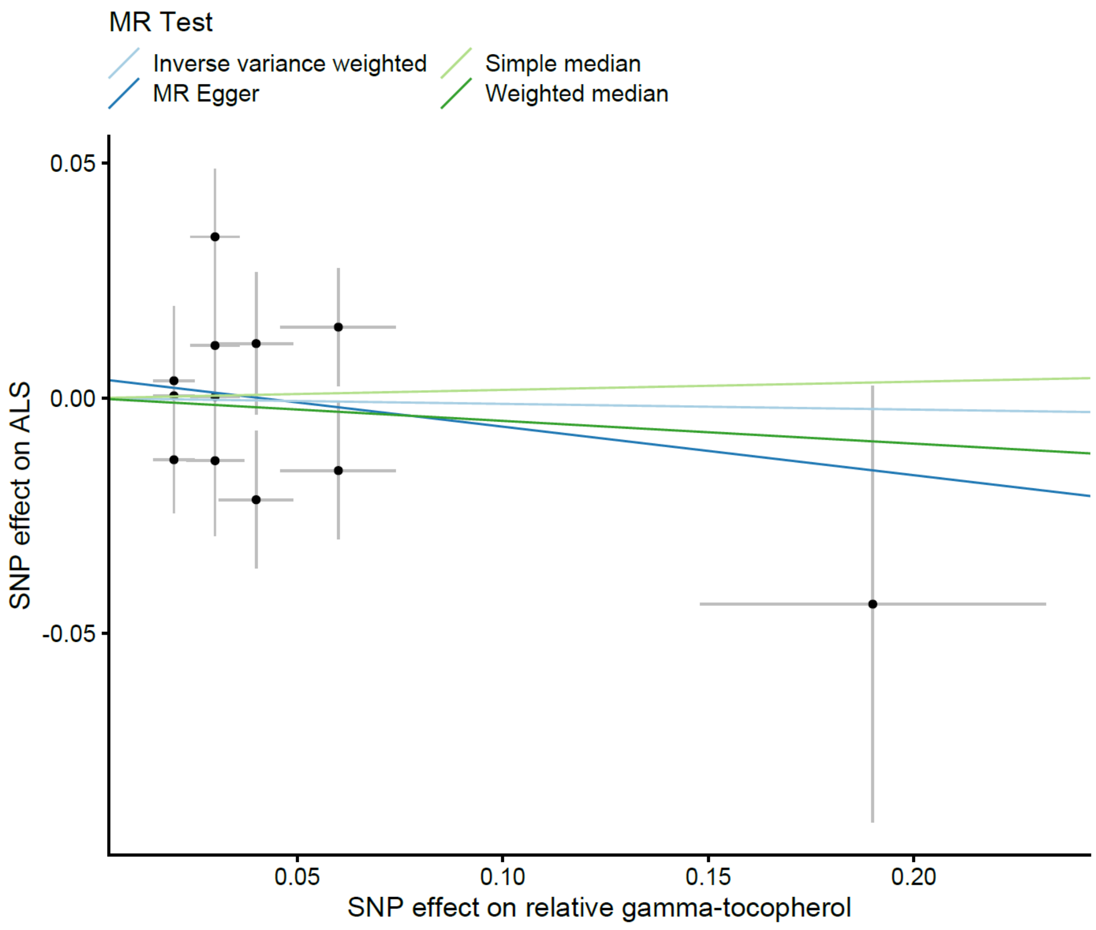

Supplemental Figure S4N. Scatterplot of relative gamma-tocopherol and amyotrophic lateral sclerosis

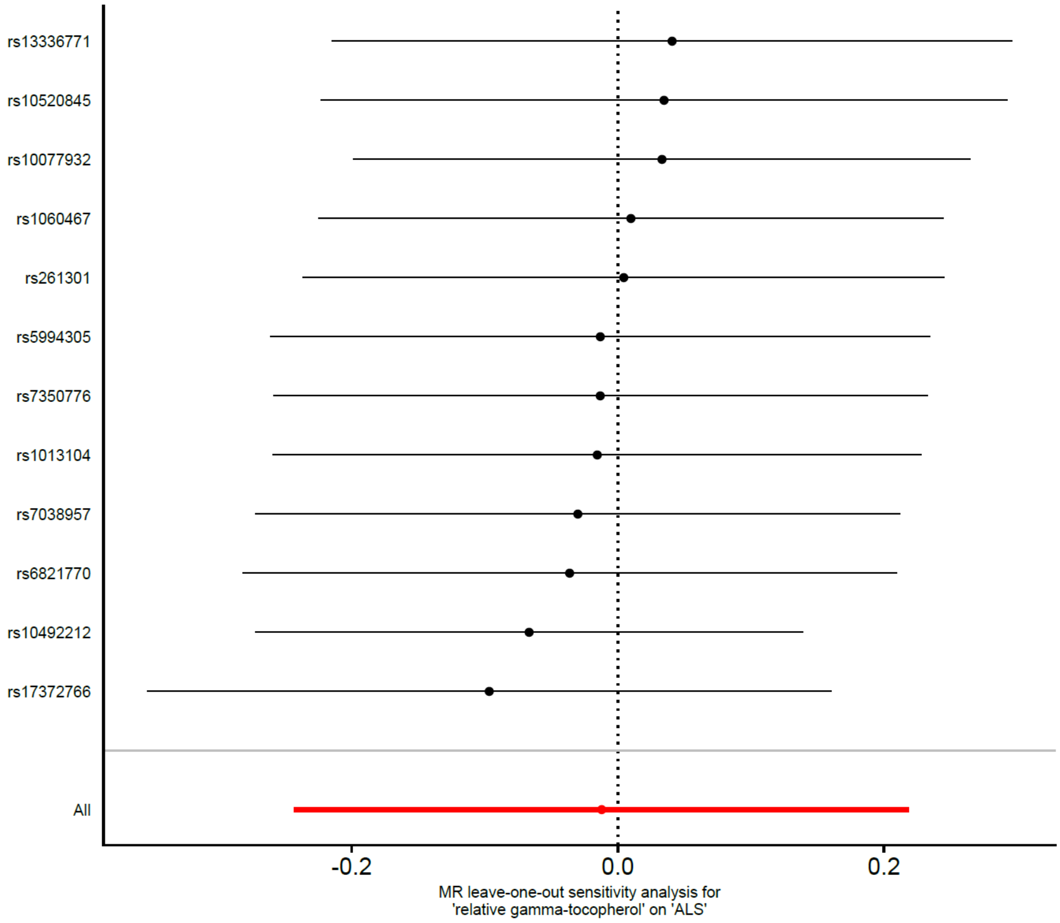

Supplemental Figure S4O. Forrest plot of relative gamma-tocopherol and amyotrophic lateral sclerosis
