# Supplementary material for: Dietary-Derived Essential Nutrients and Amyotrophic Lateral Sclerosis: A Two-Sample Mendelian Randomization Study
Source: Nutrients. 2022 Feb 22;14(5):920. doi: 10.3390/nu14050920 (PMC8912818; doi:10.3390/nu14050920)
Supplement: Supplementary file 1 [file nutrients-14-00920-s001.zip › supplementary materials/additional file1.pdf]

Dietary derived essential nutrients and amyotrophic lateral sclerosis: a two-sample mendelian randomization study

Kailin Xia[1,2,3#], Yajun Wang[1,2,3#], Linjing Zhang[1,2,3], Lu Tang[1,2,3], Gan Zhang[1,2,3], Tao Huang[4], Ninghao Huang[4], Dongsheng Fan[1,2,3*]

[1]Department of Neurology, Peking University Third Hospital, Beijing, China.

[2]Beijing Key Laboratory of Biomarker and Translational Research in Neurodegenerative Diseases, Beijing, China

[3]Key Laboratory for Neuroscience, National Health Commission/Ministry of Education, Peking University, Beijing, China

[4]Department of Epidemiology and Biostatistics, School of Public Health, Peking University, Beijing, China

#KX and YW contributed equally to this article.

*Corresponding author: Dr. Dongsheng Fan,

Department of Neurology, Peking University Third Hospital,

49 North Garden Road, Haidian District, Beijing 100191, People's Republic of China.

E-mail:dsfan2010@aliyun.com

Supplementary table S1 Characteristics of instrumental variables (IVs).

SNP	effect	SE	effect_allele	other_allele	EAF	P value	N	Traits	R2	F statistics
rs174547	0.016	0.001	C	T	0.33	4.00E-64	8631	Alpha-linolenic acid (ALA)	1.31E-02	114.681
rs174547	1.691	0.025	T	C	0.68	1.00E-200	8631	Arachidonic acid (AA)	2.31E-01	2587.593
rs16966952	0.199	0.031	G	A	0.69	2.40E-10	8631	Arachidonic acid (AA)	2.04E-03	17.661
rs1801725	0.071	0.004	T	G	0.15	8.90E-86	61054	Calcium	1.32E-03	80.444
rs1570669	0.018	0.003	G	A	0.34	9.10E-12	60966	Calcium	2.65E-04	16.161
rs1550532	0.018	0.003	C	G	0.31	8.20E-11	60998	Calcium	2.52E-04	15.404
rs7481584	0.018	0.003	G	A	0.7	1.20E-10	61011	Calcium	2.48E-04	15.123
rs780094	0.017	0.003	T	C	0.42	1.30E-10	60958	Calcium	2.57E-04	15.648
rs7336933	0.022	0.004	G	A	0.85	9.10E-10	60928	Calcium	1.27E-04	7.714
rs10491003	0.027	0.005	T	C	0.09	4.80E-09	60040	Calcium	7.96E-05	4.777
rs1801725	0.191	0.004	T	G	0.13	1.00E-200	313903	Calcium (UKB)	1.64E-03	516.596
rs838718	0.047	0.003	G	A	0.48	9.09E-73	313903	Calcium (UKB)	3.90E-04	122.573
rs1260326	0.047	0.003	T	C	0.61	3.46E-69	313903	Calcium (UKB)	3.72E-04	116.825
rs6909201	0.043	0.003	A	G	0.48	4.12E-60	313903	Calcium (UKB)	3.27E-04	102.591
rs112371897	0.071	0.004	T	C	0.91	2.54E-56	313903	Calcium (UKB)	1.64E-04	51.615
rs11078597	0.052	0.003	C	T	0.81	9.55E-54	313903	Calcium (UKB)	2.95E-04	92.503
rs6741561	0.04	0.003	C	T	0.39	1.85E-50	313903	Calcium (UKB)	2.69E-04	84.609
rs13108218	0.039	0.003	A	G	0.62	4.07E-47	313903	Calcium (UKB)	2.54E-04	79.652
rs4917	0.038	0.003	C	T	0.36	5.43E-43	313903	Calcium (UKB)	2.36E-04	73.95
rs28616221	0.046	0.003	A	G	0.82	4.53E-42	313903	Calcium (UKB)	2.21E-04	69.42
rs841572	0.033	0.003	A	G	0.59	1.30E-35	313903	Calcium (UKB)	1.86E-04	58.55
rs1688131	0.036	0.003	T	C	0.71	1.79E-35	313903	Calcium (UKB)	1.89E-04	59.31
rs2004315	0.033	0.003	T	C	0.38	5.84E-35	313903	Calcium (UKB)	1.82E-04	57.025
rs10739679	0.034	0.003	G	A	0.36	9.19E-35	313903	Calcium (UKB)	1.89E-04	59.198
rs12918968	0.032	0.003	A	C	0.44	1.06E-34	313903	Calcium (UKB)	1.79E-04	56.079
rs9530	0.032	0.003	A	G	0.55	3.64E-34	313903	Calcium (UKB)	1.79E-04	56.33
rs13107325	0.059	0.005	C	T	0.93	1.14E-32	313903	Calcium (UKB)	5.78E-05	18.13
rs6841429	0.041	0.004	C	A	0.17	1.02E-31	313903	Calcium (UKB)	9.45E-05	29.651
rs12212449	0.031	0.003	C	T	0.61	9.55E-31	313903	Calcium (UKB)	1.62E-04	50.813
rs1858800	0.032	0.003	T	C	0.65	1.61E-30	313903	Calcium (UKB)	1.65E-04	51.777
rs1177274	0.028	0.003	T	G	0.56	1.45E-26	313903	Calcium (UKB)	1.37E-04	42.934
rs12339541	0.056	0.005	C	A	0.06	1.22E-25	313903	Calcium (UKB)	4.51E-05	14.15
rs116004654	0.059	0.006	T	C	0.94	9.10E-25	313903	Calcium (UKB)	3.47E-05	10.907
rs760077	0.027	0.003	A	T	0.61	1.84E-23	313903	Calcium (UKB)	1.23E-04	38.544
rs12917235	0.027	0.003	C	T	0.37	2.33E-23	313903	Calcium (UKB)	1.20E-04	37.767
rs4758621	0.028	0.003	A	G	0.31	2.55E-22	313903	Calcium (UKB)	1.19E-04	37.27
rs7320843	0.036	0.004	C	T	0.14	2.60E-22	313903	Calcium (UKB)	6.21E-05	19.506
rs1801282	0.039	0.004	C	G	0.88	7.95E-22	313903	Calcium (UKB)	6.40E-05	20.078
rs965344	0.031	0.003	A	G	0.79	1.08E-21	313903	Calcium (UKB)	1.13E-04	35.433
rs1150781	0.044	0.005	C	G	0.91	1.92E-21	313903	Calcium (UKB)	4.04E-05	12.685
rs3931841	0.027	0.003	A	G	0.68	2.37E-21	313903	Calcium (UKB)	1.12E-04	35.255

rs34667500	0.038	0.004	A	G	0.88	3.14E-21	313903	Calcium (UKB)	6.07E-05	19.062
rs4376797	0.026	0.003	A	G	0.65	5.75E-21	313903	Calcium (UKB)	1.09E-04	34.179
rs498490	0.033	0.004	C	T	0.16	8.71E-21	313903	Calcium (UKB)	5.83E-05	18.296
rs80350997	0.046	0.005	A	G	0.92	1.03E-20	313903	Calcium (UKB)	3.97E-05	12.459
rs12519940	0.027	0.003	C	T	0.28	2.75E-20	313903	Calcium (UKB)	1.04E-04	32.662
rs848492	0.027	0.003	A	G	0.28	1.01E-19	313903	Calcium (UKB)	1.04E-04	32.662
rs7208714	0.027	0.003	G	A	0.75	1.75E-19	313903	Calcium (UKB)	9.68E-05	30.378
rs1827293	0.024	0.003	G	A	0.45	2.97E-19	313903	Calcium (UKB)	1.01E-04	31.683
rs35320690	0.026	0.003	C	T	0.72	4.71E-19	313903	Calcium (UKB)	9.65E-05	30.288
rs6734610	0.023	0.003	G	A	0.48	1.58E-18	313903	Calcium (UKB)	9.35E-05	29.344
rs7012637	0.023	0.003	A	G	0.52	5.48E-18	313903	Calcium (UKB)	9.35E-05	29.344
rs115946508	0.036	0.004	A	C	0.89	8.95E-18	313903	Calcium (UKB)	5.05E-05	15.861
rs34010237	0.03	0.004	A	G	0.84	1.39E-17	313903	Calcium (UKB)	4.82E-05	15.121
rs9388399	0.024	0.003	T	C	0.31	1.94E-17	313903	Calcium (UKB)	8.72E-05	27.381
rs4790310	0.022	0.003	C	T	0.57	3.31E-17	313903	Calcium (UKB)	8.40E-05	26.364
rs1476698	0.023	0.003	A	G	0.37	7.70E-17	313903	Calcium (UKB)	8.73E-05	27.404
rs4594967	0.023	0.003	A	G	0.67	7.83E-17	313903	Calcium (UKB)	8.28E-05	25.994
rs1875272	0.026	0.003	G	A	0.75	9.81E-17	313903	Calcium (UKB)	8.97E-05	28.169
rs11632520	0.029	0.003	C	T	0.17	1.01E-16	313903	Calcium (UKB)	8.40E-05	26.372
rs3741628	0.022	0.003	G	T	0.57	1.24E-16	313903	Calcium (UKB)	8.40E-05	26.364
rs72999033	0.043	0.005	T	C	0.93	5.12E-16	313903	Calcium (UKB)	3.07E-05	9.63
rs926103	0.022	0.003	C	T	0.35	8.02E-16	313903	Calcium (UKB)	7.80E-05	24.471
rs4633480	0.021	0.003	A	G	0.44	2.43E-15	313903	Calcium (UKB)	7.69E-05	24.149
rs1749849	0.021	0.003	C	T	0.42	2.47E-15	313903	Calcium (UKB)	7.61E-05	23.874
rs2343592	0.023	0.003	A	G	0.27	2.71E-15	313903	Calcium (UKB)	7.38E-05	23.172
rs6680117	0.027	0.003	C	T	0.18	3.20E-15	313903	Calcium (UKB)	7.62E-05	23.913
rs61770531	0.036	0.005	C	T	0.91	8.13E-15	313903	Calcium (UKB)	2.71E-05	8.492
rs9635741	0.039	0.005	C	A	0.93	8.14E-15	313903	Calcium (UKB)	2.52E-05	7.922
rs1064608	0.021	0.003	G	C	0.65	1.35E-14	313903	Calcium (UKB)	7.10E-05	22.296
rs12998379	0.026	0.003	G	A	0.19	1.43E-14	313903	Calcium (UKB)	7.37E-05	23.121
rs56397046	0.021	0.003	C	T	0.33	2.24E-14	313903	Calcium (UKB)	6.90E-05	21.669
rs62292542	0.032	0.004	G	A	0.11	2.37E-14	313903	Calcium (UKB)	3.99E-05	12.532
rs2419886	0.023	0.003	C	T	0.26	2.91E-14	313903	Calcium (UKB)	7.21E-05	22.619
rs302655	0.02	0.003	G	T	0.43	3.55E-14	313903	Calcium (UKB)	6.94E-05	21.788
rs62439474	0.022	0.003	T	C	0.7	4.81E-14	313903	Calcium (UKB)	7.20E-05	22.588
rs2520265	0.022	0.003	A	G	0.29	5.23E-14	313903	Calcium (UKB)	7.05E-05	22.147
rs7568296	0.02	0.003	T	C	0.42	5.83E-14	313903	Calcium (UKB)	6.90E-05	21.655
rs36104352	0.03	0.004	C	A	0.88	7.60E-14	313903	Calcium (UKB)	3.78E-05	11.88
rs12378991	0.036	0.005	G	A	0.08	1.08E-13	313903	Calcium (UKB)	2.43E-05	7.631
rs34372369	0.044	0.006	A	G	0.05	1.27E-13	313903	Calcium (UKB)	1.63E-05	5.109
rs12583851	0.022	0.003	T	C	0.75	1.48E-13	313903	Calcium (UKB)	6.42E-05	20.168
rs4805129	0.02	0.003	T	C	0.63	1.73E-13	313903	Calcium (UKB)	6.60E-05	20.721
rs1036332	0.022	0.003	C	A	0.26	2.09E-13	313903	Calcium (UKB)	6.59E-05	20.695
rs12922549	0.023	0.003	C	T	0.24	2.42E-13	313903	Calcium (UKB)	6.83E-05	21.443

rs1354034	0.019	0.003	T	C	0.6	3.11E-13	313903	Calcium (UKB)	6.13E-05	19.254
rs10129874	0.025	0.004	C	T	0.17	3.95E-13	313903	Calcium (UKB)	3.51E-05	11.024
rs6580981	0.019	0.003	G	A	0.46	4.69E-13	313903	Calcium (UKB)	6.35E-05	19.928
rs6719061	0.019	0.003	C	T	0.6	6.19E-13	313903	Calcium (UKB)	6.13E-05	19.254
rs6560613	0.034	0.005	C	T	0.91	6.79E-13	313903	Calcium (UKB)	2.41E-05	7.574
rs11753096	0.019	0.003	C	T	0.46	6.89E-13	313903	Calcium (UKB)	6.35E-05	19.928
rs62362239	0.02	0.003	C	T	0.67	7.53E-13	313903	Calcium (UKB)	6.26E-05	19.654
rs8034835	0.019	0.003	A	G	0.47	9.95E-13	313903	Calcium (UKB)	6.37E-05	19.985
rs13254847	0.023	0.003	C	T	0.22	1.33E-12	313903	Calcium (UKB)	6.43E-05	20.174
rs11187838	0.019	0.003	G	A	0.43	1.37E-12	313903	Calcium (UKB)	6.26E-05	19.664
rs17774672	0.025	0.004	G	A	0.16	2.09E-12	313903	Calcium (UKB)	3.34E-05	10.5
rs36086195	0.018	0.003	T	C	0.42	3.78E-12	313903	Calcium (UKB)	5.59E-05	17.54
rs900399	0.019	0.003	G	A	0.6	3.79E-12	313903	Calcium (UKB)	6.13E-05	19.254
rs10917386	0.019	0.003	T	C	0.31	8.49E-12	313903	Calcium (UKB)	5.47E-05	17.16
rs6118	0.03	0.004	C	T	0.9	8.86E-12	313903	Calcium (UKB)	3.23E-05	10.125
rs12613807	0.018	0.003	C	T	0.56	9.55E-12	313903	Calcium (UKB)	5.65E-05	17.742
rs7864156	0.018	0.003	G	T	0.61	1.42E-11	313903	Calcium (UKB)	5.46E-05	17.13
rs17164683	0.02	0.003	C	T	0.27	1.66E-11	313903	Calcium (UKB)	5.58E-05	17.521
rs4925104	0.018	0.003	G	T	0.47	1.90E-11	313903	Calcium (UKB)	5.71E-05	17.936
rs12974855	0.026	0.004	A	G	0.86	1.97E-11	313903	Calcium (UKB)	3.24E-05	10.174
rs7559013	0.026	0.004	C	A	0.87	2.07E-11	313903	Calcium (UKB)	3.04E-05	9.557
rs3026445	0.018	0.003	T	C	0.37	2.52E-11	313903	Calcium (UKB)	5.35E-05	16.784
rs2636695	0.021	0.003	G	T	0.77	2.74E-11	313903	Calcium (UKB)	5.53E-05	17.357
rs11629876	0.018	0.003	C	T	0.33	2.93E-11	313903	Calcium (UKB)	5.07E-05	15.92
rs11777067	0.02	0.003	T	C	0.77	3.90E-11	313903	Calcium (UKB)	5.01E-05	15.743
rs2448036	0.029	0.004	G	T	0.11	4.28E-11	313903	Calcium (UKB)	3.28E-05	10.292
rs6894167	0.017	0.003	C	T	0.52	5.07E-11	313903	Calcium (UKB)	5.11E-05	16.031
rs9420589	0.017	0.003	T	G	0.56	6.74E-11	313903	Calcium (UKB)	5.04E-05	15.825
rs10224210	0.019	0.003	C	T	0.72	6.87E-11	313903	Calcium (UKB)	5.15E-05	16.174
rs72847071	0.03	0.005	G	A	0.09	7.17E-11	313903	Calcium (UKB)	1.88E-05	5.897
rs611150	0.022	0.003	T	C	0.82	9.18E-11	313903	Calcium (UKB)	5.06E-05	15.876
rs4976647	0.018	0.003	C	A	0.67	1.03E-10	313903	Calcium (UKB)	5.07E-05	15.92
rs57564578	0.033	0.005	G	A	0.07	1.08E-10	313903	Calcium (UKB)	1.81E-05	5.672
rs7221118	0.021	0.003	T	C	0.21	1.18E-10	313903	Calcium (UKB)	5.18E-05	16.259
rs62134669	0.024	0.004	C	T	0.85	1.22E-10	313903	Calcium (UKB)	2.92E-05	9.18
rs12932755	0.017	0.003	A	G	0.51	1.62E-10	313903	Calcium (UKB)	5.11E-05	16.05
rs7968405	0.022	0.003	C	T	0.82	2.24E-10	313903	Calcium (UKB)	5.06E-05	15.876
rs1495747	0.019	0.003	C	T	0.28	2.36E-10	313903	Calcium (UKB)	5.15E-05	16.174
rs77722590	0.023	0.004	A	G	0.85	2.38E-10	313903	Calcium (UKB)	2.69E-05	8.431
rs3130618	0.021	0.003	C	A	0.8	2.50E-10	313903	Calcium (UKB)	5.00E-05	15.681
rs507666	0.021	0.003	G	A	0.19	2.69E-10	313903	Calcium (UKB)	4.80E-05	15.083
rs697852	0.022	0.003	G	A	0.82	3.13E-10	313903	Calcium (UKB)	5.06E-05	15.876
rs11730491	0.022	0.004	T	G	0.83	3.20E-10	313903	Calcium (UKB)	2.72E-05	8.537
rs56230940	0.031	0.005	T	C	0.93	3.20E-10	313903	Calcium (UKB)	1.59E-05	5.005

rs61594679	0.018	0.003	C	T	0.29	4.23E-10	313903	Calcium (UKB)	4.72E-05	14.825
rs17668044	0.018	0.003	A	G	0.71	4.66E-10	313903	Calcium (UKB)	4.72E-05	14.825
rs9806062	0.026	0.004	G	T	0.89	5.10E-10	313903	Calcium (UKB)	2.64E-05	8.273
rs6722613	0.016	0.003	G	A	0.57	5.31E-10	313903	Calcium (UKB)	4.44E-05	13.944
rs6590227	0.025	0.004	C	T	0.12	5.42E-10	313903	Calcium (UKB)	2.63E-05	8.25
rs7313874	0.017	0.003	T	C	0.6	6.13E-10	313903	Calcium (UKB)	4.91E-05	15.414
rs6013892	0.032	0.005	A	C	0.93	6.19E-10	313903	Calcium (UKB)	1.70E-05	5.333
rs218671	0.016	0.003	G	T	0.55	6.44E-10	313903	Calcium (UKB)	4.49E-05	14.081
rs3798236	0.017	0.003	T	C	0.37	7.93E-10	313903	Calcium (UKB)	4.77E-05	14.971
rs10444863	0.02	0.003	T	C	0.21	1.02E-09	313903	Calcium (UKB)	4.70E-05	14.747
rs13389219	0.016	0.003	C	T	0.39	1.02E-09	313903	Calcium (UKB)	4.31E-05	13.534
rs11117777	0.022	0.004	C	T	0.16	1.62E-09	313903	Calcium (UKB)	2.59E-05	8.131
rs2807880	0.02	0.003	C	T	0.18	1.87E-09	313903	Calcium (UKB)	4.18E-05	13.12
rs35674179	0.026	0.004	C	A	0.9	2.00E-09	313903	Calcium (UKB)	2.42E-05	7.605
rs2100431	0.017	0.003	A	C	0.3	3.04E-09	313903	Calcium (UKB)	4.30E-05	13.487
rs35758545	0.016	0.003	C	T	0.35	3.10E-09	313903	Calcium (UKB)	4.12E-05	12.943
rs722298	0.016	0.003	A	G	0.56	3.38E-09	313903	Calcium (UKB)	4.47E-05	14.018
rs11746728	0.016	0.003	C	T	0.65	4.74E-09	313903	Calcium (UKB)	4.12E-05	12.943
rs1262217	0.02	0.003	A	G	0.17	4.99E-09	313903	Calcium (UKB)	4.00E-05	12.543
rs11792928	0.017	0.003	C	T	0.29	5.27E-09	313903	Calcium (UKB)	4.21E-05	13.224
rs872629	0.024	0.004	A	C	0.89	6.00E-09	313903	Calcium (UKB)	2.25E-05	7.072
rs12933677	0.015	0.003	T	C	0.47	6.97E-09	313903	Calcium (UKB)	3.97E-05	12.455
rs1604081	0.022	0.004	C	T	0.14	7.93E-09	313903	Calcium (UKB)	2.32E-05	7.284
rs8011945	0.026	0.005	G	T	0.09	1.14E-08	313903	Calcium (UKB)	1.41E-05	4.429
rs8081353	0.025	0.004	C	T	0.1	1.21E-08	313903	Calcium (UKB)	2.24E-05	7.031
rs3794695	0.019	0.003	T	C	0.81	1.21E-08	313903	Calcium (UKB)	3.93E-05	12.347
rs681664	0.018	0.003	C	T	0.22	1.27E-08	313903	Calcium (UKB)	3.94E-05	12.356
rs61649210	0.015	0.003	G	A	0.45	1.35E-08	313903	Calcium (UKB)	3.94E-05	12.375
rs35587941	0.015	0.003	T	G	0.57	1.37E-08	313903	Calcium (UKB)	3.90E-05	12.255
rs56313825	0.015	0.003	A	G	0.6	1.44E-08	313903	Calcium (UKB)	3.82E-05	12
rs6830950	0.017	0.003	T	C	0.27	1.50E-08	313903	Calcium (UKB)	4.03E-05	12.659
rs12675477	0.017	0.003	T	C	0.73	1.61E-08	313903	Calcium (UKB)	4.03E-05	12.659
rs11122848	0.015	0.003	A	G	0.47	1.67E-08	313903	Calcium (UKB)	3.97E-05	12.455
rs5751350	0.016	0.003	A	G	0.67	1.75E-08	313903	Calcium (UKB)	4.01E-05	12.579
rs2785171	0.015	0.003	G	A	0.61	1.76E-08	313903	Calcium (UKB)	3.79E-05	11.895
rs2585135	0.015	0.003	A	G	0.54	1.85E-08	313903	Calcium (UKB)	3.96E-05	12.42
rs10898822	0.015	0.003	A	G	0.51	1.91E-08	313903	Calcium (UKB)	3.98E-05	12.495
rs11218725	0.015	0.003	G	A	0.62	2.03E-08	313903	Calcium (UKB)	3.75E-05	11.78
rs308981	0.023	0.004	C	T	0.12	2.25E-08	313903	Calcium (UKB)	2.22E-05	6.983
rs55754498	0.033	0.006	C	T	0.05	2.37E-08	313903	Calcium (UKB)	9.15E-06	2.874
rs2918247	0.017	0.003	A	G	0.23	2.48E-08	313903	Calcium (UKB)	3.62E-05	11.374
rs12794834	0.015	0.003	C	T	0.52	3.08E-08	313903	Calcium (UKB)	3.98E-05	12.48
rs10754439	0.015	0.003	T	G	0.58	3.78E-08	313903	Calcium (UKB)	3.88E-05	12.18
rs7913072	0.021	0.004	G	A	0.86	3.95E-08	313903	Calcium (UKB)	2.11E-05	6.637

rs2855799	0.018	0.003	G	A	0.81	4.06E-08	313903	Calcium (UKB)	3.53E-05	11.081
rs113911787	0.017	0.003	A	G	0.77	4.13E-08	313903	Calcium (UKB)	3.62E-05	11.374
rs76758508	0.015	0.003	C	T	0.32	4.19E-08	313903	Calcium (UKB)	3.47E-05	10.88
rs2245715	0.024	0.004	A	G	0.1	4.45E-08	313903	Calcium (UKB)	2.06E-05	6.48
rs35852840	0.031	0.006	A	C	0.94	4.70E-08	313903	Calcium (UKB)	9.59E-06	3.011
rs2769264	0.313	0.034	G	T	0.16	2.63E-20	2603	Copper	8.75E-03	22.964
rs1175550	0.198	0.032	G	A	0.22	5.03E-10	2603	Copper	5.05E-03	13.196
rs174547	0.355	0.014	C	T	0.33	2.63E-151	8631	Dihomo-gamma-linolenic acid (DGLA)	3.49E-02	312.125
rs16966952	0.22	0.013	G	A	0.69	7.55E-65	8631	Dihomo-gamma-linolenic acid (DGLA)	1.42E-02	124.712
rs174546	-0.128	0.012	T	C	0.403	4.81E-24	13499	Docosahexaenoic acid (DHA)	3.73E-03	50.479
rs261334	-0.11	0.015	C	G	0.769	1.44E-13	13498	Docosahexaenoic acid (DHA)	1.47E-03	19.869
rs143988316	-0.15	0.024	T	C	0.069	1.10E-09	13494	Docosahexaenoic acid (DHA)	3.64E-04	4.911
rs2281591	-0.108	0.018	G	A	0.134	3.66E-09	13498	Docosahexaenoic acid (DHA)	6.11E-04	8.245
rs11604424	-0.083	0.014	T	C	0.757	7.84E-09	13495	Docosahexaenoic acid (DHA)	9.29E-04	12.551
rs174547	0.075	0.003	T	C	0.67	4.00E-154	8631	Docosapentaenoic acid (DPA)	3.20E-02	285.451
rs3734398	0.04	0.003	C	T	0.43	1.00E-43	8631	Docosapentaenoic acid (DPA)	1.01E-02	88.015
rs780094	0.017	0.003	T	C	0.41	9.00E-09	8631	Docosapentaenoic acid (DPA)	1.80E-03	15.56
rs174538	0.083	0.005	G	A	0.72	5.00E-58	8631	Eicosapentaenoic acid (EPA)	1.29E-02	112.529
rs3798713	0.035	0.005	C	G	0.43	2.00E-12	8631	Eicosapentaenoic acid (EPA)	2.78E-03	24.081
rs174547	0.016	0.001	T	C	0.67	2.29E-72	8631	Gamma linolenic acid (GLA)	1.54E-02	134.902
rs16966952	0.006	0.001	G	A	0.69	5.05E-11	8631	Gamma linolenic acid (GLA)	2.28E-03	19.693
rs855791	0.181	0.007	G	A	0.55	1.32E-139	48972	Iron	6.76E-03	333.191
rs1800562	0.328	0.016	A	G	0.07	2.72E-97	48972	Iron	1.12E-03	54.776
rs1799945	0.189	0.01	G	C	0.15	1.10E-81	48972	Iron	1.86E-03	91.255
rs1260326	-0.086	0.01	C	T	0.639	9.75E-18	22549	Isoleucine	1.52E-03	34.376
rs1440580	0.073	0.009	A	T	0.481	1.68E-14	24772	Isoleucine	1.20E-03	29.763
rs17789027	0.109	0.01	G	A	0.384	6.16E-30	24725	Leucine	2.48E-03	61.454
rs1260326	-0.08	0.01	C	T	0.639	1.07E-15	22500	Leucine	1.32E-03	29.797
rs12325419	-0.082	0.015	A	G	0.12	4.55E-08	22500	Leucine	2.82E-04	6.345
rs99780	0.148	0.012	T	C	0.401	3.35E-32	13523	Linoleic acid (LA)	5.03E-03	68.318
rs964184	-0.193	0.017	C	G	0.856	1.14E-28	13525	Linoleic acid (LA)	2.28E-03	30.882
rs7412	-0.295	0.028	T	C	0.057	3.40E-25	13523	Linoleic acid (LA)	8.71E-04	11.79
rs1800588	0.129	0.014	T	C	0.249	7.41E-19	13525	Linoleic acid (LA)	2.21E-03	29.915
rs769449	0.142	0.017	A	G	0.157	8.04E-17	13524	Linoleic acid (LA)	1.38E-03	18.627
rs10402112	-0.171	0.021	A	T	0.1	7.90E-16	13522	Linoleic acid (LA)	8.73E-04	11.819
rs17414716	-0.3	0.038	G	A	0.028	3.26E-15	13522	Linoleic acid (LA)	2.58E-04	3.488
rs79225634	0.096	0.013	T	C	0.352	1.66E-13	13523	Linoleic acid (LA)	1.86E-03	25.195
rs12239737	-0.1	0.014	A	T	0.26	1.20E-12	13523	Linoleic acid (LA)	1.46E-03	19.745
rs34232196	-0.253	0.035	T	C	0.042	1.30E-12	13526	Linoleic acid (LA)	3.01E-04	4.076
rs174418	-0.086	0.013	C	T	0.563	9.42E-12	13524	Linoleic acid (LA)	1.71E-03	23.227
rs1260326	-0.082	0.013	C	T	0.637	1.28E-10	13527	Linoleic acid (LA)	1.43E-03	19.426
rs4296389	-0.081	0.013	T	C	0.326	6.37E-10	13521	Linoleic acid (LA)	1.26E-03	17.045
rs144064722	0.23	0.04	G	A	0.026	7.45E-09	13520	Linoleic acid (LA)	1.28E-04	1.736
rs76366838	0.287	0.05	A	G	0.018	9.06E-09	13524	Linoleic acid (LA)	8.99E-05	1.215

rs17231506	0.084	0.015	T	C	0.252	1.43E-08	13523	Linoleic acid (LA)	9.09E-04	12.307
rs144723570	-0.332	0.06	T	C	0.011	3.42E-08	13523	Linoleic acid (LA)	5.05E-05	0.682
rs9804646	-0.106	0.019	T	C	0.124	4.05E-08	13520	Linoleic acid (LA)	4.93E-04	6.663
rs2863979	0.014	0.002	A	G	0.724	1.44E-17	7812	lysine	3.62E-03	28.367
rs4072037	0.01	0.001	T	C	0.54	2.01E-36	23829	Magnesium	2.08E-03	49.78
rs10858939	0.007	0.001	A	C	0.71	1.05E-16	23829	Magnesium	8.47E-04	20.194
rs3925584	0.006	0.001	T	C	0.55	5.20E-16	23829	Magnesium	7.48E-04	17.832
rs11144134	0.011	0.001	C	T	0.08	8.21E-15	23829	Magnesium	7.47E-04	17.823
rs13146355	0.005	0.001	A	G	0.44	6.27E-13	23829	Magnesium	5.17E-04	12.325
rs448378	0.004	0.001	A	G	0.53	1.25E-08	23829	Magnesium	3.35E-04	7.973
rs320485	0.009	0.002	T	C	0.132	4.97E-08	7795	methionine	9.01E-04	7.029
rs2731672	0.095	0.012	C	T	0.74	3.85E-16	20436	Phenylalanine	1.26E-03	25.712
rs1718309	-0.077	0.01	G	A	0.603	2.50E-15	22660	Phenylalanine	1.33E-03	30.23
rs4253238	0.067	0.01	T	C	0.555	2.53E-11	20435	Phenylalanine	1.08E-03	22.153
rs1697421	0.05	0.005	A	G	0.49	1.14E-27	21726	Phosphorus	2.30E-03	50.091
rs947583	0.035	0.005	C	T	0.29	3.45E-12	21733	Phosphorus	9.28E-04	20.195
rs9469578	0.059	0.009	C	T	0.92	1.11E-11	21734	Phosphorus	2.91E-04	6.327
rs17265703	0.036	0.006	A	G	0.85	4.32E-09	21733	Phosphorus	4.22E-04	9.183
rs2970818	0.047	0.008	A	T	0.09	4.38E-09	21734	Phosphorus	2.60E-04	5.655
rs921943	0.207	0.016	T	C	0.29	1.90E-39	9639	Selenium	7.15E-03	69.409
rs6586282	-0.113	0.019	T	C	0.85	3.96E-09	9639	Selenium	9.36E-04	9.026
rs13122250	0.006	0.001	T	C	0.554	8.95E-12	7804	Tryotophan	3.00E-03	23.514
rs6901004	-0.006	0.001	C	G	0.574	1.08E-11	7804	Tryotophan	2.88E-03	22.522
rs1016522	0.006	0.001	A	G	0.58	1.59E-10	7804	Tryotophan	2.59E-03	20.277
rs4306882	-0.006	0.001	T	G	0.615	2.52E-10	7804	Tryotophan	2.43E-03	19.031
rs6935961	-0.006	0.001	A	G	0.558	3.75E-10	7804	Tryotophan	2.45E-03	19.141
rs284191	-0.006	0.001	A	G	0.615	1.97E-09	7804	Tryotophan	2.18E-03	17.081
rs7463805	-0.005	0.001	T	C	0.521	4.60E-09	7804	Tryotophan	2.22E-03	17.342
rs1559063	0.005	0.001	C	G	0.621	7.82E-09	7804	Tryotophan	2.01E-03	15.743
rs4695138	0.005	0.001	A	T	0.535	8.00E-09	7804	Tryotophan	2.13E-03	16.64
rs38271	-0.005	0.001	A	G	0.593	1.19E-08	7804	Tryotophan	1.99E-03	15.533
rs2111118	0.005	0.001	T	C	0.621	1.21E-08	7804	Tryotophan	1.94E-03	15.145
rs603446	0.005	0.001	T	C	0.445	1.38E-08	7804	Tryotophan	2.03E-03	15.888
rs972459	0.005	0.001	T	C	0.579	1.97E-08	7804	Tryotophan	1.93E-03	15.075
rs4958379	-0.005	0.001	A	G	0.433	2.18E-08	7804	Tryotophan	1.94E-03	15.181
rs1373962	0.005	0.001	T	C	0.597	2.71E-08	7804	Tryotophan	1.90E-03	14.872
rs9511152	-0.005	0.001	A	G	0.559	2.99E-08	7804	Tryotophan	1.95E-03	15.243
rs710580	-0.005	0.001	A	C	0.357	3.57E-08	7804	Tryotophan	1.81E-03	14.185
rs7584842	-0.005	0.001	T	C	0.538	4.15E-08	7804	Tryotophan	1.97E-03	15.371
rs6480970	-0.005	0.001	A	G	0.616	4.29E-08	7804	Tryotophan	1.80E-03	14.046
rs4615256	0.005	0.001	A	G	0.533	4.99E-08	7804	Tryotophan	1.89E-03	14.782
rs9637599	0.114	0.009	C	A	0.47	1.67E-35	24899	Valine	3.10E-03	77.36
rs10211524	0.086	0.009	A	G	0.41	5.24E-20	24898	Valine	1.63E-03	40.703
rs7406661	0.079	0.013	C	T	0.243	5.35E-10	22659	Valine	6.27E-04	14.224

rs7655059	-0.068	0.011	G	C	0.218	8.91E-10	24897	Valine	5.14E-04	12.815
rs2072560	0.105	0.018	C	T	0.929	3.28E-09	24895	Valine	1.87E-04	4.654
rs2120019	0.287	0.033	T	C	0.79	1.55E-18	2603	Zinc	9.64E-03	25.321
rs1532423	0.178	0.026	A	G	0.37	6.40E-12	2603	Zinc	8.39E-03	22.019
rs577185477	-0.379	0.01	C	T	0.015	1.00E-200	443734	absolute 25OHD	1.02E-04	45.371
rs11723621	-0.187	0.002	G	A	0.291	1.00E-200	443734	absolute 25OHD	7.22E-03	3229.247
rs10832289	-0.069	0.002	T	A	0.41	1.00E-200	443734	absolute 25OHD	1.33E-03	589.157
rs12803256	0.1	0.002	G	A	0.771	1.00E-200	443734	absolute 25OHD	1.48E-03	659.244
rs212100	0.06	0.003	T	C	0.16	1.77E-110	443734	absolute 25OHD	3.01E-04	133.786
rs10859995	-0.039	0.002	C	T	0.581	7.03E-89	443734	absolute 25OHD	4.38E-04	194.557
rs6127099	-0.037	0.002	T	A	0.279	9.30E-62	443734	absolute 25OHD	2.49E-04	110.667
rs12123821	0.074	0.005	T	C	0.048	2.25E-59	443734	absolute 25OHD	5.43E-05	24.1
rs7680709	-0.029	0.002	G	A	0.455	6.97E-49	443734	absolute 25OHD	2.41E-04	107.112
rs964184	0.04	0.003	C	G	0.864	5.11E-44	443734	absolute 25OHD	1.03E-04	45.638
rs2011425	-0.046	0.004	G	T	0.079	9.66E-38	443734	absolute 25OHD	5.43E-05	24.104
rs1800588	-0.03	0.002	T	C	0.215	2.65E-36	443734	absolute 25OHD	1.20E-04	53.372
rs8018720	-0.032	0.003	C	G	0.82	4.04E-36	443734	absolute 25OHD	1.05E-04	46.442
rs2762942	0.053	0.004	A	G	0.942	7.99E-35	443734	absolute 25OHD	3.73E-05	16.537
rs261291	-0.022	0.002	C	T	0.356	2.89E-28	443734	absolute 25OHD	1.26E-04	55.73
rs7828742	-0.022	0.002	G	A	0.597	3.06E-28	443734	absolute 25OHD	1.32E-04	58.45
rs1629122	-0.022	0.002	A	C	0.644	3.96E-26	443734	absolute 25OHD	1.16E-04	51.297
rs3750296	-0.021	0.002	C	G	0.341	2.09E-24	443734	absolute 25OHD	1.05E-04	46.745
rs6123359	0.032	0.003	G	A	0.105	7.74E-24	443734	absolute 25OHD	4.30E-05	19.079
rs780094	0.018	0.002	C	T	0.617	6.41E-19	443734	absolute 25OHD	8.41E-05	37.327
rs58542926	0.032	0.004	T	C	0.076	8.57E-19	443734	absolute 25OHD	2.48E-05	10.986
rs1972994	-0.018	0.002	T	A	0.647	7.99E-18	443734	absolute 25OHD	7.61E-05	33.783
rs1800775	-0.017	0.002	A	C	0.486	1.56E-17	443734	absolute 25OHD	8.18E-05	36.293
rs7528419	0.019	0.002	G	A	0.225	2.41E-16	443734	absolute 25OHD	5.28E-05	23.445
rs2229742	-0.026	0.003	C	G	0.104	7.13E-16	443734	absolute 25OHD	2.73E-05	12.125
rs2608953	0.022	0.003	C	T	0.835	1.41E-15	443734	absolute 25OHD	3.96E-05	17.566
rs11264360	0.018	0.002	A	T	0.243	3.34E-15	443734	absolute 25OHD	5.14E-05	22.826
rs73015021	0.023	0.003	G	A	0.121	1.15E-14	443734	absolute 25OHD	2.86E-05	12.687
rs157595	-0.016	0.002	G	A	0.614	2.95E-14	443734	absolute 25OHD	6.17E-05	27.38
rs1149605	0.019	0.003	C	T	0.171	7.34E-14	443734	absolute 25OHD	3.58E-05	15.903
rs7412	0.027	0.004	T	C	0.082	8.32E-14	443734	absolute 25OHD	1.89E-05	8.398
rs17765311	-0.015	0.002	C	A	0.345	1.35E-13	443734	absolute 25OHD	5.58E-05	24.744
rs3768013	-0.015	0.002	A	G	0.37	1.37E-13	443734	absolute 25OHD	5.75E-05	25.514
rs1229984	-0.047	0.006	C	T	0.973	4.85E-13	443734	absolute 25OHD	6.17E-06	2.736
rs2847500	-0.021	0.003	A	G	0.124	7.79E-13	443734	absolute 25OHD	2.52E-05	11.183
rs1011468	-0.014	0.002	A	G	0.476	1.35E-12	443734	absolute 25OHD	5.65E-05	25.068
rs2909218	0.017	0.002	T	C	0.793	2.81E-12	443734	absolute 25OHD	3.61E-05	16.041
rs2074735	0.027	0.004	C	G	0.064	6.55E-12	443734	absolute 25OHD	1.28E-05	5.666
rs12317268	-0.019	0.003	G	A	0.152	9.15E-12	443734	absolute 25OHD	2.70E-05	11.962
rs1047891	-0.014	0.002	A	C	0.316	1.16E-11	443734	absolute 25OHD	4.49E-05	19.919

rs62007299	-0.014	0.002	A	G	0.709	1.69E-11	443734	absolute 25OHD	4.21E-05	18.679
rs7519574	0.017	0.003	A	G	0.182	2.09E-11	443734	absolute 25OHD	3.01E-05	13.349
rs58073039	-0.014	0.002	G	A	0.298	2.16E-11	443734	absolute 25OHD	4.23E-05	18.766
rs867772	-0.014	0.002	G	A	0.682	3.64E-11	443734	absolute 25OHD	4.28E-05	19.004
rs1858889	0.013	0.002	C	A	0.501	3.85E-11	443734	absolute 25OHD	4.92E-05	21.845
rs804280	0.013	0.002	A	C	0.582	4.43E-11	443734	absolute 25OHD	4.76E-05	21.125
rs111529171	-0.015	0.002	C	G	0.216	6.24E-11	443734	absolute 25OHD	3.27E-05	14.495
rs7569755	0.014	0.002	A	G	0.292	8.03E-11	443734	absolute 25OHD	3.94E-05	17.483
rs6724965	-0.017	0.003	G	A	0.172	1.29E-10	443734	absolute 25OHD	2.65E-05	11.751
rs10887718	-0.012	0.002	T	C	0.527	1.44E-10	443734	absolute 25OHD	4.62E-05	20.49
rs77924615	-0.016	0.002	A	G	0.198	1.46E-10	443734	absolute 25OHD	2.94E-05	13.035
rs12997242	-0.013	0.002	A	G	0.438	2.23E-10	443734	absolute 25OHD	4.47E-05	19.817
rs56044892	0.015	0.002	T	C	0.211	2.85E-10	443734	absolute 25OHD	2.98E-05	13.229
rs523583	0.012	0.002	C	A	0.469	5.58E-10	443734	absolute 25OHD	4.32E-05	19.159
rs10500209	-0.013	0.002	C	T	0.282	6.18E-10	443734	absolute 25OHD	3.49E-05	15.5
rs34726834	0.014	0.002	T	C	0.254	6.65E-10	443734	absolute 25OHD	3.25E-05	14.432
rs6698680	-0.012	0.002	G	A	0.464	8.99E-10	443734	absolute 25OHD	4.21E-05	18.671
rs2037511	0.016	0.003	A	G	0.165	9.29E-10	443734	absolute 25OHD	2.33E-05	10.344
rs6438900	0.014	0.002	G	C	0.261	9.59E-10	443734	absolute 25OHD	3.25E-05	14.42
rs8091117	-0.024	0.004	A	C	0.065	1.03E-09	443734	absolute 25OHD	1.03E-05	4.556
rs35733741	0.013	0.002	A	G	0.42	1.15E-09	443734	absolute 25OHD	4.07E-05	18.045
rs57631352	-0.013	0.002	G	A	0.297	1.48E-09	443734	absolute 25OHD	3.44E-05	15.273
rs7718395	0.013	0.002	G	C	0.32	1.67E-09	443734	absolute 25OHD	3.56E-05	15.795
rs532436	-0.015	0.003	A	G	0.184	2.17E-09	443734	absolute 25OHD	2.43E-05	10.772
rs960596	0.012	0.002	T	C	0.34	2.23E-09	443734	absolute 25OHD	3.61E-05	16.039
rs8103262	0.013	0.002	C	T	0.305	3.18E-09	443734	absolute 25OHD	3.35E-05	14.868
rs10818769	-0.017	0.003	G	C	0.857	3.35E-09	443734	absolute 25OHD	1.93E-05	8.573
rs8063706	0.013	0.002	T	A	0.273	3.64E-09	443734	absolute 25OHD	3.11E-05	13.812
rs78649910	-0.018	0.003	A	T	0.11	4.32E-09	443734	absolute 25OHD	1.52E-05	6.751
rs6773343	0.013	0.002	T	C	0.72	5.20E-09	443734	absolute 25OHD	3.10E-05	13.748
rs7301806	0.012	0.002	A	G	0.471	5.38E-09	443734	absolute 25OHD	3.82E-05	16.965
rs964184	0.04	0.01	G	C	0.15	7.80E-12	7781	absolute alpha-tocopherol	5.24E-04	4.081
rs2108622	0.03	0.01	T	C	0.21	1.40E-10	7781	absolute alpha-tocopherol	3.84E-04	2.987
rs11057830	0.03	0.01	A	G	0.15	8.20E-09	7781	absolute alpha-tocopherol	2.95E-04	2.295
rs6564851	0.149	0.015	G	T	0.395	1.60E-24	3881	absolute beta-carotene	1.22E-02	47.715
rs7680948	-0.19	0.03	A	C	0.2	4.97E-09	441	absolute lycopene	2.91E-02	13.16
rs10882272	-0.03	0.004	C	T	0.35	6.51E-15	5006	absolute retinol	5.11E-03	25.715
rs1667255	0.03	0.004	C	A	0.31	6.35E-14	5006	absolute retinol	4.81E-03	24.17
rs10245705	-0.07	0.013	T	C	0.02	1.95E-07	7276	relative alpha-tocopherol	1.64E-04	1.191
rs7238006	0.03	0.006	T	C	0.93	6.77E-07	7276	relative alpha-tocopherol	4.96E-04	3.607
rs11145330	0.03	0.007	A	C	0.89	1.95E-06	7276	relative alpha-tocopherol	5.24E-04	3.812
rs2074731	-0.02	0.004	A	C	0.17	2.31E-06	7276	relative alpha-tocopherol	1.02E-03	7.427
rs1404410	-0.02	0.005	C	G	0.79	4.57E-06	7276	relative alpha-tocopherol	6.75E-04	4.91
rs1532701	0.01	0.003	A	G	0.55	5.07E-06	7276	relative alpha-tocopherol	7.56E-04	5.503

rs261342	-0.02	0.004	C	G	0.79	5.41E-06	7276	relative alpha-tocopherol	1.33E-03	9.705
rs11992435	0.03	0.007	A	G	0.95	6.38E-06	7276	relative alpha-tocopherol	2.27E-04	1.649
rs7930821	0.07	0.015	T	C	0.02	7.53E-06	7276	relative alpha-tocopherol	1.19E-04	0.865
rs10163969	-0.04	0.008	T	G	0.04	9.39E-06	7276	relative alpha-tocopherol	2.64E-04	1.92
rs10935814	-0.04	0.008	A	G	0.1	9.44E-06	7276	relative alpha-tocopherol	5.75E-04	4.182
rs1060467	0.02	0.005	A	G	0.59	2.61E-07	5822	relative gamma-tocopherol	1.64E-03	9.569
rs5994305	0.03	0.006	A	G	0.83	7.15E-07	5822	relative gamma-tocopherol	1.13E-03	6.612
rs261301	0.03	0.007	T	C	0.13	2.06E-06	5822	relative gamma-tocopherol	7.56E-04	4.404
rs1013104	-0.02	0.005	T	C	0.44	3.83E-06	5822	relative gamma-tocopherol	1.67E-03	9.747
rs7350776	0.02	0.005	C	G	0.7	3.86E-06	5822	relative gamma-tocopherol	1.07E-03	6.218
rs7038957	-0.03	0.006	T	C	0.83	3.86E-06	5822	relative gamma-tocopherol	1.13E-03	6.612
rs10077932	-0.04	0.009	T	C	0.14	4.08E-06	5822	relative gamma-tocopherol	8.74E-04	5.093
rs10520845	0.19	0.042	A	C	0.02	5.27E-06	5822	relative gamma-tocopherol	1.38E-04	0.806
rs13336771	0.06	0.014	A	C	0.17	7.39E-06	5822	relative gamma-tocopherol	9.03E-04	5.261
rs10492212	-0.03	0.006	T	C	0.16	8.66E-06	5822	relative gamma-tocopherol	1.08E-03	6.298
rs6821770	0.04	0.009	A	G	0.14	8.92E-06	5822	relative gamma-tocopherol	8.95E-04	5.212
rs17372766	0.06	0.014	T	C	0.16	9.56E-06	5822	relative gamma-tocopherol	8.13E-04	4.734
rs58411567	-0.21	0.05	A	G	0.216	3.02E-07	1957	relative retinol	3.05E-03	5.979
rs1176744	-0.21	0.05	C	A	0.318	3.51E-07	1957	relative retinol	3.91E-03	7.677
rs945817	-0.28	0.05	A	G	0.192	6.46E-07	1957	relative retinol	4.98E-03	9.784
rs1842947	-0.19	0.04	G	A	0.522	8.34E-07	1957	relative retinol	5.75E-03	11.314
rs2417325	0.33	0.08	T	C	0.933	1.29E-06	1957	relative retinol	1.09E-03	2.126
rs149478645	-0.51	0.14	G	A	0.024	1.30E-06	1957	relative retinol	3.18E-04	0.622
rs7926028	-0.13	0.04	T	G	0.445	2.75E-06	1957	relative retinol	2.67E-03	5.226
rs17005512	-0.22	0.06	C	G	0.17	2.77E-06	1957	relative retinol	1.94E-03	3.798
rs3898702	-0.22	0.05	T	C	0.2	3.02E-06	1957	relative retinol	3.16E-03	6.197
rs9586119	0.35	0.08	C	T	0.073	3.34E-06	1957	relative retinol	1.32E-03	2.585
rs149113848	-0.96	0.27	G	C	0.01	3.47E-06	1957	relative retinol	8.85E-05	0.173
rs75308833	-0.49	0.15	T	C	0.022	3.51E-06	1957	relative retinol	2.40E-04	0.469
rs10019071	0.66	0.16	A	G	0.019	3.64E-06	1957	relative retinol	3.24E-04	0.634
rs6550239	-0.18	0.05	A	G	0.742	4.40E-06	1957	relative retinol	2.53E-03	4.967
rs138139857	0.37	0.11	T	C	0.039	4.46E-06	1957	relative retinol	4.32E-04	0.844
rs112293959	-0.43	0.13	G	A	0.031	5.70E-06	1957	relative retinol	3.38E-04	0.66
rs114515641	0.41	0.13	G	T	0.03	7.12E-06	1957	relative retinol	2.96E-04	0.578
rs1472234	0.14	0.04	G	C	0.384	8.56E-06	1957	relative retinol	2.96E-03	5.804
rs2147337	0.16	0.04	G	T	0.66	9.00E-06	1957	relative retinol	3.67E-03	7.2
rs2367816	0.23	0.05	G	A	0.771	9.46E-06	1957	relative retinol	3.81E-03	7.486
rs4135385	0.21	0.05	G	A	0.237	9.80E-06	1957	relative retinol	3.26E-03	6.387
rs118025446	-0.48	0.12	A	G	0.031	9.84E-06	1957	relative retinol	4.90E-04	0.958
rs602662	0.16	0.008	A	G	0.6	4.10E-96	37283	absolute vitamin B-12	5.15E-03	192.984
rs34324219	0.21	0.011	C	A	0.88	8.80E-71	37283	absolute vitamin B-12	2.06E-03	77.13
rs1801222	0.11	0.007	G	A	0.59	1.10E-52	37283	absolute vitamin B-12	3.20E-03	119.847
rs1131603	0.19	0.017	C	T	0.06	4.30E-28	37283	absolute vitamin B-12	3.78E-04	14.095
rs41281112	0.17	0.016	C	T	0.95	9.60E-27	37283	absolute vitamin B-12	2.88E-04	10.727

rs1141321	0.061	0.007	C	T	0.63	1.40E-16	37283	absolute vitamin B-12	9.50E-04	35.434
rs1256335	0.14	0.02	A	G	0.79	1.40E-15	4763	absolute vitamin B-6	3.41E-03	16.307
rs33972313	0.36	0.018	C	T	0.968	4.61E-90	52018	absolute ascorbate	4.76E-04	24.792
rs13028225	0.102	0.009	T	C	0.857	2.38E-30	52018	absolute ascorbate	6.05E-04	31.5
rs2559850	0.058	0.006	A	G	0.598	6.30E-20	52018	absolute ascorbate	8.64E-04	44.964
rs117885456	0.078	0.012	A	G	0.087	1.70E-11	52018	absolute ascorbate	1.29E-04	6.713
rs6693447	0.039	0.006	T	G	0.551	6.25E-10	52018	absolute ascorbate	4.02E-04	20.913
rs56738967	0.041	0.007	C	G	0.321	7.62E-10	52018	absolute ascorbate	2.87E-04	14.958
rs10051765	0.039	0.007	C	T	0.342	3.64E-09	52018	absolute ascorbate	2.69E-04	13.974
rs1130214	0.04	0.007	A	C	0.283	1.33E-08	52018	absolute ascorbate	2.55E-04	13.254
rs174547	0.036	0.007	C	T	0.328	3.84E-08	52018	absolute ascorbate	2.24E-04	11.662
rs11167905	0.0804	0.0164	T	C	0.8545	9.83E-07	2085	relative ascorbate	2.87E-03	5.988
rs13069990	-0.0506	0.011	T	C	0.3781	4.44E-06	2085	relative ascorbate	4.77E-03	9.989
rs13103690	-0.0474	0.0104	T	G	0.5359	5.20E-06	2085	relative ascorbate	4.96E-03	10.374
rs2070006	0.0512	0.0112	T	C	0.3713	4.76E-06	2085	relative ascorbate	4.68E-03	9.793
rs577596	-0.0567	0.0114	A	G	0.3267	6.68E-07	2085	relative ascorbate	5.22E-03	10.929
rs6713914	0.0593	0.0116	T	C	0.5741	3.22E-07	2085	relative ascorbate	6.13E-03	12.846
rs6826474	-0.1383	0.0288	T	C	0.0393	1.56E-06	2085	relative ascorbate	8.35E-04	1.741
rs6834631	0.1306	0.0267	T	G	0.9596	1.03E-06	2085	relative ascorbate	8.90E-04	1.855
rs7112460	0.1084	0.0223	T	C	0.0658	1.14E-06	2085	relative ascorbate	1.39E-03	2.906
rs8057559	0.1395	0.0314	T	C	0.0312	9.10E-06	2085	relative ascorbate	5.72E-04	1.193
rs808686	0.0598	0.0128	A	G	0.6086	3.01E-06	2085	relative ascorbate	4.99E-03	10.44
rs8105491	-0.0698	0.0148	T	G	0.154	2.30E-06	2085	relative ascorbate	2.78E-03	5.806

Abbreviations: EAF: effect allele frequency; se: standard error; UKB: UK Biobank
